# New perspectives on Neanderthal dispersal and turnover from Stajnia Cave (Poland)

**DOI:** 10.1038/s41598-020-71504-x

**Published:** 2020-09-08

**Authors:** Andrea Picin, Mateja Hajdinjak, Wioletta Nowaczewska, Stefano Benazzi, Mikołaj Urbanowski, Adrian Marciszak, Helen Fewlass, Marjolein D. Bosch, Paweł Socha, Krzysztof Stefaniak, Marcin Żarski, Andrzej Wiśniewski, Jean-Jacques Hublin, Adam Nadachowski, Sahra Talamo

**Affiliations:** 1 Department of Human Evolution, Max Planck Institute for Evolutionary Anthropology, Deutscher Platz 6, 04103 Leipzig, Germany; 2 Department of Evolutionary Genetics, Max Planck Institute for Evolutionary Anthropology, Deutscher Platz 6, 04103 Leipzig, Germany; 3 Department of Human Biology, Wrocław University, ul. Kuźnicza 35, 50-138, Wrocław, Poland; 4 Department of Cultural Heritage, University of Bologna, Via degli Ariani 1, 48121 Ravenna, Italy; 5 Independent Researcher, Warsaw, Poland; 6 Department of Palaeozoology, Institute of Environmental Biology, Wrocław University, Sienkiewicza 21, 50-335 Wrocław, Poland; 7 McDonald Institute for Archaeological Research, University of Cambridge, Downing Street, CB2 3ER, Cambridge, UK; 8 Division of Palaeozoology, Department of Evolutionary Biology and Ecology, Wrocław University, ul. Sienkiewicza 21, 50-335 Wrocław, Poland; 9 Polish Geological Institute, National Research Institute, Rakowiecka 4, 00975 Warsaw, Poland; 10 Institute of Archaeology, University of Wrocław, Szewska 48, 50-139, Wrocław, Poland; 11 Collège de France, 11 Place Marcellin Berthelot, 75005 Paris, France; 12 Institute of Systematics and Evolution of Animals, Polish Academy of Sciences, Sławkowska 17, 31-016, Kraków, Poland; 13 Department of Chemistry G. Ciamician, University of Bologna, Via Selmi 2, 40126 Bologna, Italy

**Keywords:** Archaeology, Evolutionary genetics, Palaeontology

## Abstract

The Micoquian is the broadest and longest enduring cultural facies of the Late Middle Palaeolithic that spread across the periglacial and boreal environments of Europe between Eastern France, Poland, and Northern Caucasus. Here, we present new data from the archaeological record of Stajnia Cave (Poland) and the paleogenetic analysis of a Neanderthal molar S5000, found in a Micoquian context. Our results demonstrate that the mtDNA genome of Stajnia S5000 dates to MIS 5a making the tooth the oldest Neanderthal specimen from Central-Eastern Europe. Furthermore, S5000 mtDNA has the fewest number of differences to mtDNA of Mezmaiskaya 1 Neanderthal from Northern Caucasus, and is more distant from almost contemporaneous Neanderthals of Scladina and Hohlenstein-Stadel. This observation and the technological affinity between Poland and the Northern Caucasus could be the result of increased mobility of Neanderthals that changed their subsistence strategy for coping with the new low biomass environments and the increased foraging radius of gregarious animals. The Prut and Dniester rivers were probably used as the main corridors of dispersal. The persistence of the Micoquian techno-complex in South-Eastern Europe infers that this axis of mobility was also used at the beginning of MIS 3 when a Neanderthal population turnover occurred in the Northern Caucasus.

## Introduction

Reconstructing the adaptability and behavioural plasticity of archaic humans to ecological changes and faunal turnovers has been a major goal in paleoanthropology and an increasing number of investigations over the past decade have revealed complex scenarios of hominin migrations, interbreeding, and extinctions^1–3^. During the Pleistocene, climatic deteriorations and the extension of the Scandinavian ice sheet in the Northern hemisphere caused demographic decline across the mid-latitude territories of Europe^4,5^. A broad consensus suggests that, rather than tracking favourable habitats to the south, archaic humans demised during the harsh glacial stages, and, only after an increase of the average temperatures, new groups from refuge zones repopulated the northern areas^4^. This model is in close accordance with phylogeographical studies of several vegetal and faunal taxa indicating postglacial colonisation from Iberia, Italy, the Balkans, and western Asia^6^. However, which of these glacial refugia had major roles in the human repopulation processes during the Pleistocene and which routes were followed in resettling the northern territories is still little known.

Since late Marine Isotope Stage (MIS) 9, Neanderthals occupied the western regions most affected by climatic fluctuations and the occupational hiatuses documented in Northwestern and Central Europe during the glacial cycles indicates recurrent episodes of recolonisation^7–10^. An important climatic change that altered the habitat of Neanderthals in the northern territories occurred during the Last Glacial (MIS 5d—MIS 3) after the warm forested environment of the Eemian (MIS 5e) shifted to more open steppe/taiga habitats favouring the migration of cold adapted fauna from the Arctic (e.g. woolly mammoth, woolly rhino, reindeer)^11^. The abrupt drop of the temperatures and the increased aridity caused a demographic contraction in Central-Eastern Europe and, only during the climatic ameliorations of the interpleniglacial periods (MIS 5c, MIS 5a, and MIS 3), Neanderthals returned to the regions above 48° N latitude^12^. The new ecological settings and the expansion of the territory of migratory species fostered Neanderthals to develop novel strategies for coping with resource acquisitions in xeric grassland. In Central Europe and the Eastern European Plains, Neanderthals enhanced the common flake based toolkits with different types of asymmetric bifacial tools, leaf-shaped artefacts and bifacial scrapers^13,14^. This new techno-complex is generally known as Micoquian (or *Keilmessergruppen* in the German literature) and is documented in a vast area from the Saône River to the western shore of the Caspian Sea. Generally, the evidences from Germany to Poland with fringes in Hungary and north-eastern France are named Central European Micoquian whereas the examples from the eastern Carpathians and the Lower Volga are classified as Eastern Micoquian. Even though the settlements of these territories have been intermittent due to the recurrence of climatic deteriorations, the production of Micoquian stone tools lasted from the MIS 5c/MIS 5a to the end of the Middle Palaeolithic^13–16^. This technological continuity is restricted to the *Mammuthus-Coelodonta* biome and is absent in the regions facing the Mediterranean suggesting that this new technical behaviour permitted greater adaptive flexibility to the low biomass and the extreme seasonality of the boreal environment.

Recently, genetic studies on human fossils identified a population turnover of Altai Neanderthals by western European Neanderthals at ~ 90 ka^1^. This dispersal is contemporaneous with the emergence of Micoquian bifacial tools in Central and Eastern Europe (SI Sect. 1)^13^, and their spread in the Altai region is recorded at Chagyrskaya Cave^17,18^. After MIS 4, a second population turnover occurred in the Caucasus where the Neanderthal fossil of Mezmaiskaya 2 (MIS 3) shares significantly more derived alleles with other late western Neanderthals than with the local group of Mezmaiskaya 1 (MIS 4)^19^. However, this replacement was not followed by a technological break because the Micoquian techno-complex persisted in the region from late MIS 5 to the final Middle Palaeolithic^20,21^.

These genetic results spotlight that the two major demographic turnover events in Neanderthal history are associated with the Micoquian cultural tradition. Adding new archaeological and genetic data from fossils associated with this techno-complex will be pivotal for a deeper understanding of Neanderthal’s adaptive flexibility and mobility in steppe/taiga habitats during the Middle Palaeolithic. From this perspective, the territory of Poland is of crucial importance because of its geographical position at the crossroads between the Western European Plains and the Urals, and between Central Europe and the south-eastern territories. Thus far, there are very few Neanderthal remains associated with the Micoquian, and genetic information has only been extracted from samples of Feldhofer Cave^22,23^, Mezmaiskaya Cave^19,24–26^ and Hohlenstein-Stadel^27,28^ (Fig. S4).

**Figure 1:**
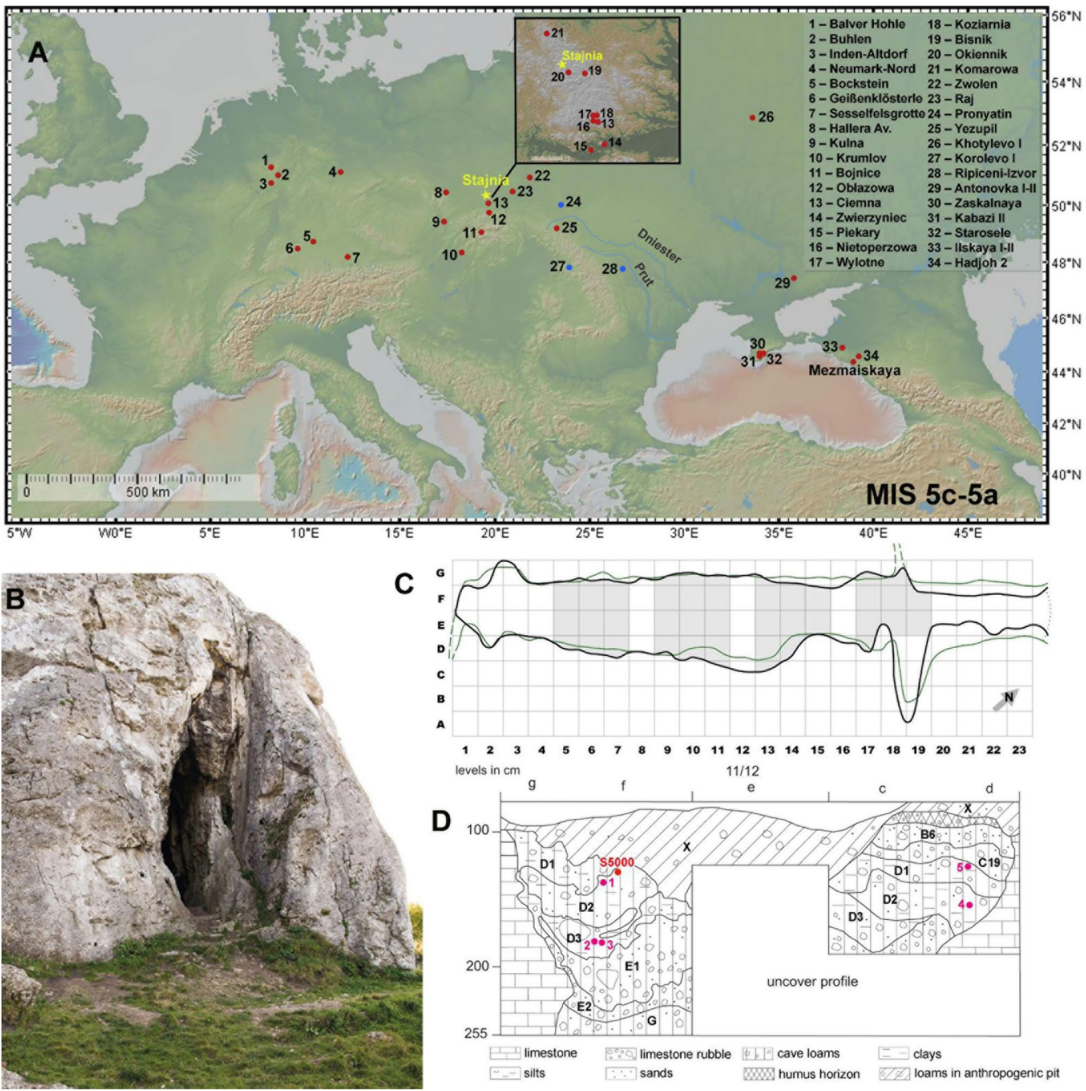
(**A**) Map showing the location of the Micoquian (red circle) and Levallois-Mousterian (blue circle) sites in Europe during MIS 5c—5a (base map from GeoMappApp (www.geomapapp.org)); (**B**) Stajnia Cave; (**C**) Planimetry of Stajnia Cave; (**D**) Stratigraphic sequence of squares 11/12: 1 – U-Th sample W1400-1417 (52,000 +1,900/−1,700 BP, 52,000 + 500/-200 BP); 2—^14^C sample S-EVA 27812 (> 49,000 ^14^C BP); 3—^14^C sample S-EVA 27814 (> 49,000 ^14^C BP); 4—^14^C sample S-EVA 27823 (> 49,000 ^14^C BP); 5—^14^C sample S-EVA 27827 (44,590 ± 690 ^14^C BP).

Here, we present new results from the archaeological record of Stajnia Cave, and the mitochondrial DNA (mtDNA) of a Neanderthal molar (S5000). Stajnia Cave is located at 359 m a.s.l. (50°36′58"N, 19°29′04"E) between the villages Mirów and Bobolice in the Kraków-Częstochowa Upland (Poland) (Fig. 1B). The cave is a rocky elevation on the Upper Oxfordian massive limestone with a narrow morphology (length of ~ 23 m, width of ~ 2–4 m, and height ~ 6 m) (Fig. 1C). Archaeological fieldwork at the site was carried out between 2007 and 2010, and the excavation covered an extension of ~ 16 m^2^ in the rear of the cave. The ~ 1.5 m stratigraphic sequence is complicated due to post-depositional frost disturbances, partial sediment sinking, and modern distortions^29^. The cave loam is divided into 15 lithostratigraphic layers accumulated between MIS 5c and MIS 1 (SI Sect. 2). The faunal assemblage is dominated by cold adapted species (SI Sect. 5) whereas the lithic assemblages of units E and D are distinctive of the Central European Micoquian. Stajnia Cave yielded the discovery of three Neanderthal teeth^30–32^ and, aside from a fragment of a mandibular incisor from Ciemna Cave^33^, dated to the MIS 3, they are the only examples of human remains attributed to the Middle Palaeolithic in Poland.

## Results

Radiocarbon dates on five animal bone samples reveal that layers E1, D3 and D2, are older than 49,000 years BP. These new radiocarbon dates are in agreement with two U/Th dates of ~ 52,900 BP on mammoth teeth from layer D2b^29^ and with previous studies^29^ associating layer E1 with MIS 4, and layers D3 and D2 with early MIS 3 (see SI Sect. 2 and Table S1 for more info). One date from layer D1 range from 47,610 to 46,130 cal BP at 68.2% probability (see Table S1).

**Figure 2:**
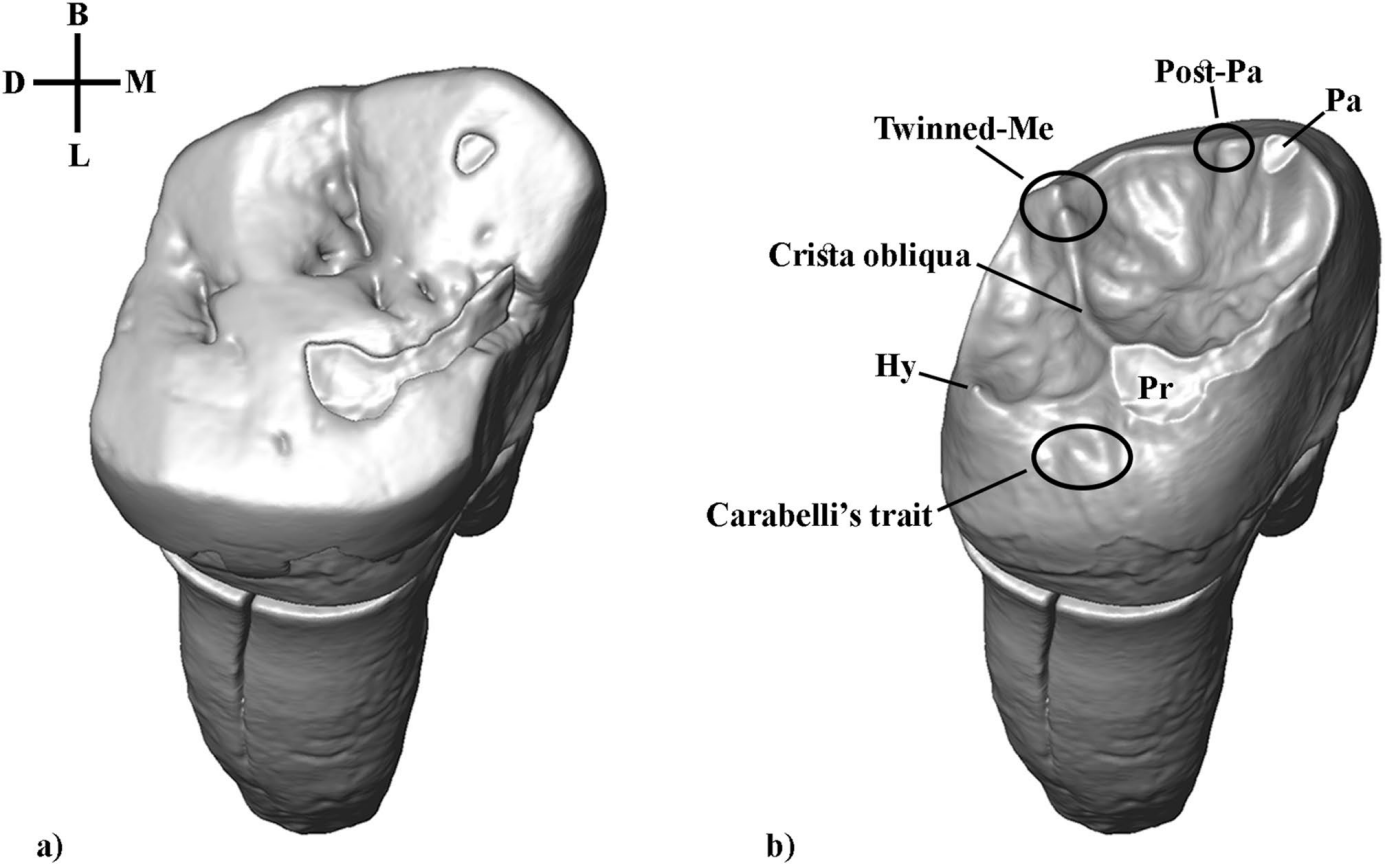
S5000 (RM^2^): 3D digital model of the reconstructed crown (**a**) and enamel-dentine junction (EDJ, **b**). Pa, Paracone; Me, Metacone; Pr, protocone; Hy, Hypocone.

In 2007, a human tooth was discovered in layer D2. Morphological description (SI Sect. 3)^32^, and morphometric data both attribute the Stajnia S5000 tooth to Neanderthal. The enamel-dentine junction (EDJ) surface of Stajnia S5000 is well preserved, except for the dentine horn tip of the paracone and the horn tip of the protocone, both affected by tooth wear (Fig. 2). The trigon and talon basins exhibit several accessory ridges running from both the dentine horns and marginal ridges towards the centre of the basins. The tooth lacks parastyle expression, but it shows a Carabelli’s trait (grade 5). Moreover, S5000 presents a post-paracone tubercle of minor expression, and a Type II crista obliqua pattern, i.e. between the protocone and the metacone, both usually observed in Neanderthal M^2^s^34,35^. Noteworthy, EDJ surface exhibits twinned dentine horns on the metacone, similar in size and shape. Up to know, such trait was observed in seven Neanderthal M^1^s and two Neanderthal M^3^s, but not previously in the M^2^^35^. We attempted to directly date the Neanderthal tooth S5000. Unfortunately, the resulting age (MAMS-40506, 22,480 ± 70 ^14^C BP) is an underestimate of the real age due to contamination by modern carbon, most probably caused by the presence of glue/preservatives applied post-excavation (see Materials and Methods, Table S1).

The Neanderthal tooth was also sampled for genetic analysis. Between 24.4 and 97.0% of the sequences recovered from DNA extracts of Stajnia S5000 matched the Neanderthal state (Table S4) at positions in the mitochondrial genome at which Neanderthals and modern humans differ from each other^36^. After restricting the analysis to putatively deaminated DNA fragments, i.e. that show terminal C to T substitutions relative to the reference genome^36^, the support for the Neanderthal state increased to between 84.7% and 100%, indicating that the specimen contains ancient mtDNA fragments of Neanderthal origin as well as some residual present-day human DNA contamination, which cannot be entirely eliminated from the data.

Although the average coverage of the mtDNA was 363-fold, five positions were covered by two or fewer sequences and nine positions had less than two thirds of sequences supporting the same state, which were not resolved even after the realignment of the sequences to the Neanderthal mtDNA (Table S5). To investigate this further, we separated putative Neanderthal and present-day human sequences in silico based on their sharing of the human or Neanderthal state at ‘diagnostic’ positions^37^. We detected high frequencies of C to T substitutions among the Neanderthal sequences and much lower signals of deamination among the ones assigned to present-day humans (Table S6). Nevertheless, C to T substitution frequencies among the present-day human-like sequences are 7.0 and 4.6%, respectively, indicating that the human contamination is more heavily damaged than in most other—but not all^37,38^—ancient hominin material studied previously. The lower consensus support for nine unresolved positions can thus be explained by residual present-day human contamination in the fraction of putatively deaminated sequences. Thus, we excluded these positions from all downstream analyses relating Stajnia S5000 mtDNA to the mtDNA genomes of Neanderthals, Denisovans and present-day humans.

**Figure 3:**
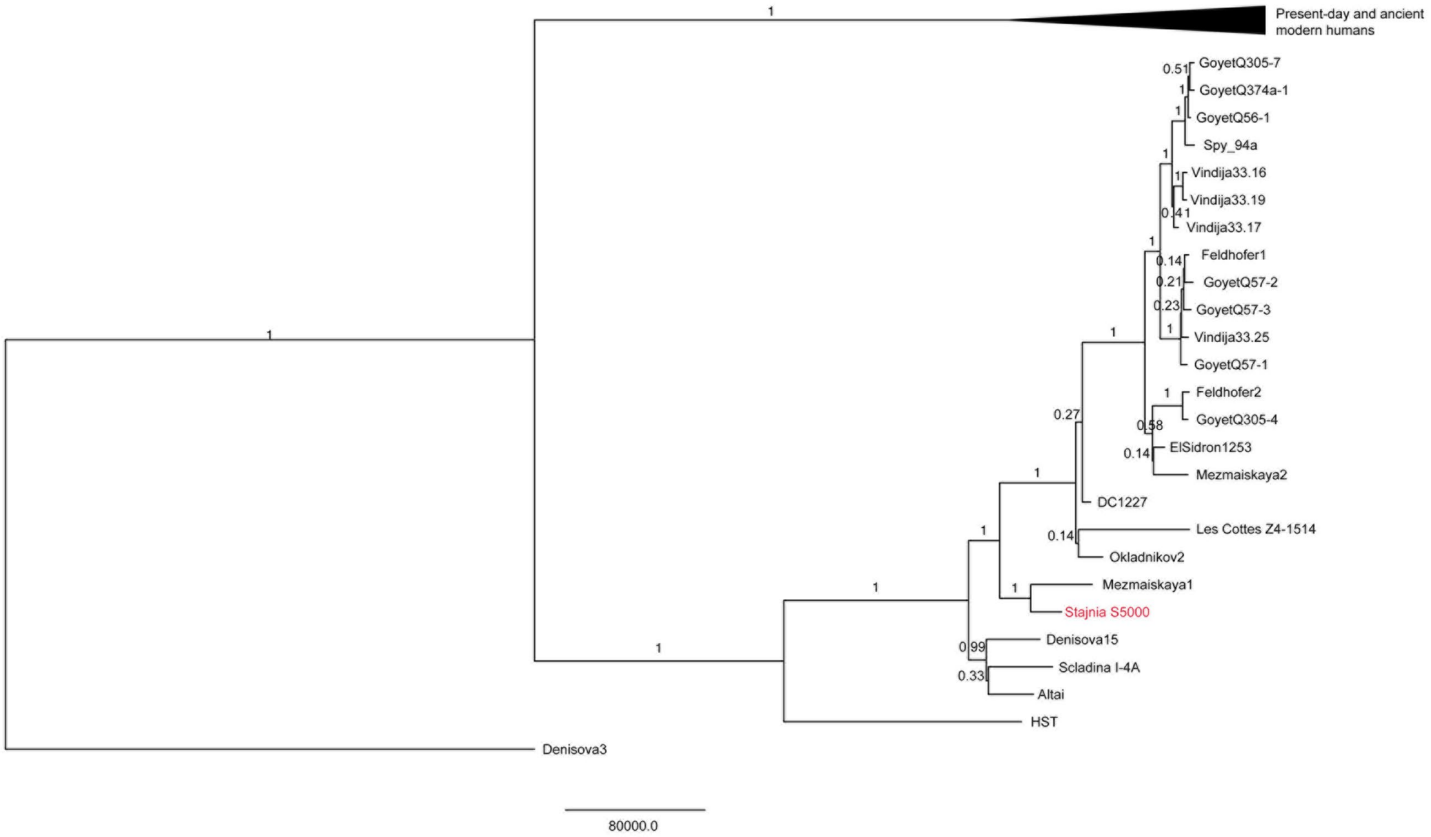
Bayesian phylogenetic tree relating the mitochondrial genome of Stajnia S5000 to the mitochondrial genomes of 24 Neanderthals, 54 present-day humans and ten ancient modern humans. Only coding region was used to reconstruct this tree. The mtDNA of Stajnia S5000 is indicated in red and the branches leading to the mtDNAs of present-day and ancient modern humans are collapsed for visualization purposes. The posterior probabilities are indicated above the branches. The mtDNA of Denisova 3 was used to root the tree.

The mitochondrial genome of Stajnia S5000 falls within the known variation of Neanderthals (Fig. 3, S2, S3). Both the Bayesian tree and the Maximum Parsimony trees show that the mtDNA of Stajnia S5000 falls close to that of Mezmaiskaya 1 Neanderthal from the Caucasus, with both of them falling outside of the mtDNA variation of the later European Neanderthals (Fig. 3, S2, S3). Furthermore, we estimated that the mtDNA genome of Stajnia S5000 dates to ~ 116 ka according to the branch length of the mtDNA tree, albeit with large confidence intervals (95% HPDI: 83,101–152,515 years ago).

A total of 13,500 vertebrate fossils were discovered between Unit E and Unit A. The faunal assemblage is dominated by cold adapted species such as reindeer, steppe wisent, woolly mammoth and woolly rhino whereas the carnivore paleocommunity is composed of cave bears, red foxes, wolves, and Polar foxes (SI Sect. 5, Table S9). Although taphonomic and archeozoological analysis has not yet been performed, the deposition of at least part of the collection of larger mammals bones occurred as a consequence of the human activity.

**Figure 4:**
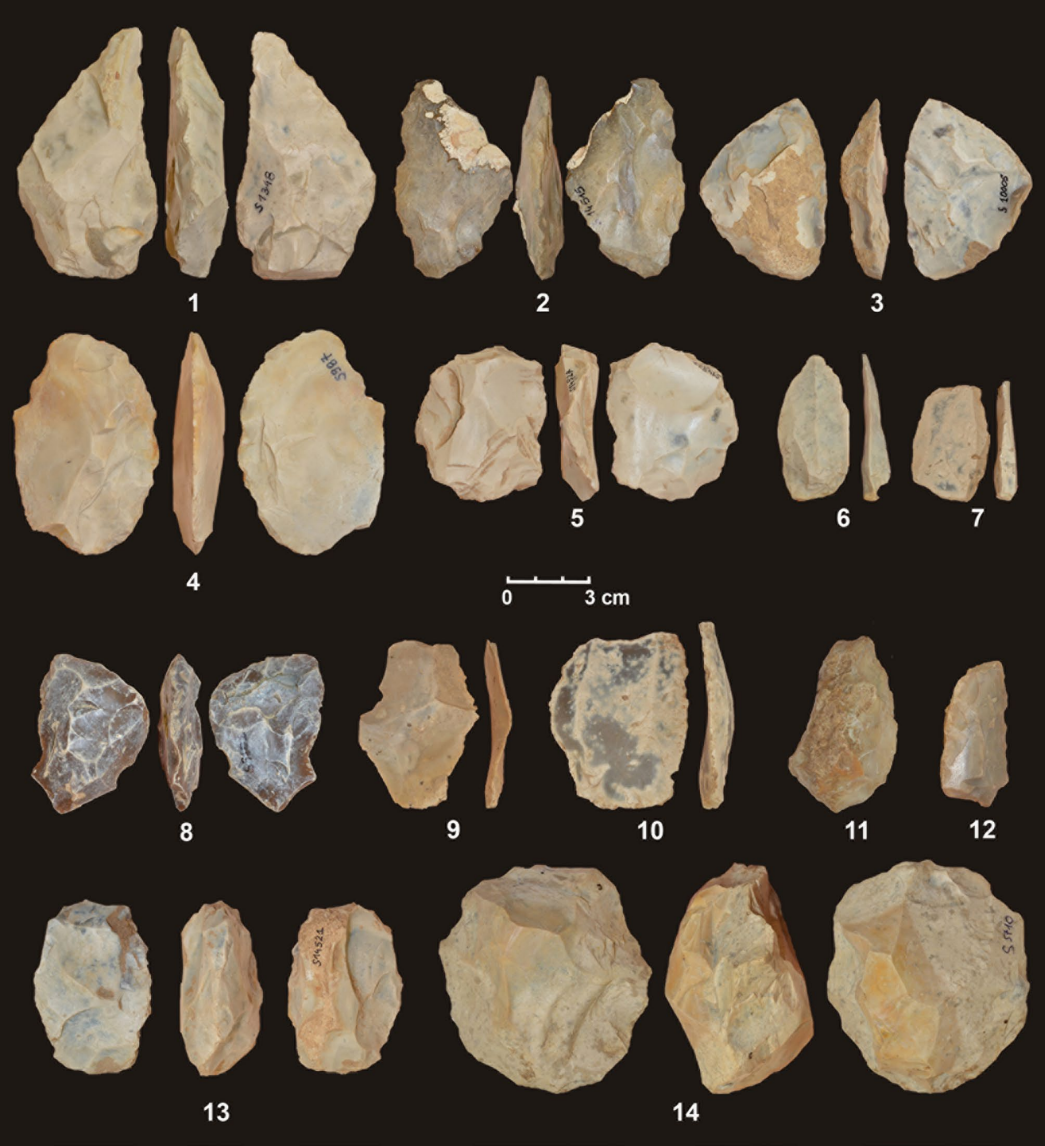
Lithic artefacts from Stajnia Cave: Layer D1—1–3) bifacial tool, 4, 5) preform of bifacial tool; 6, 7, 10) Levallois recurrent unidirectional flake, 8) fragment of bifacial tool; 11–12) scraper; 13) exhausted discoid core. Layer D2—9) Levallois recurrent centripetal flakes. Layer E1—14) discoid core.

The lithic assemblages of unit D and unit E of Stajnia Cave are produced on Jurassic flint gathered from outcrops located in the neighbourhood of the site. The analysis reveals a low number of cortical items and flakes resulting from the managing of the core convexities (Tables S10–S11) indicating that, inside the cave, the knapping activities were limited and most of the artefacts entered the site as part of the toolkit. The main concept of flake production is based on the centripetal exploitation of the core volume (Table S10). In the assemblage, within exhausted discoid cores (Fig. 4 no. 13–14), hierarchised centripetal cores are common whereas the Levallois recurrent centripetal method is attested only in one example in layer D2. The other Levallois cores found in layers D1 and D3 underwent severe taphonomic processes making the determination of the modality applied difficult.

According to this main concept of reduction, centripetal artefacts are frequent whereas core–edge removal flakes and pseudo-Levallois points are present at a lower frequency (Table S10). In layers D1 and D2, some Levallois flakes are detected. These blanks were produced using the Levallois modalities recurrent unidirectional, bidirectional and centripetal (Table S10, Fig. 4 no. 6, 7, 9, 10). In other examples, the Levallois flakes are undetermined due to the taphonomic processes that polished the dorsal surfaces making the reconstruction of the patterns of the knapping exploitation difficult. The remaining artefacts of the secondary operative chains are hierarchised bidirectional, simple unidirectional and polyhedral cores. These reduction sequences were aimed at the production of ordinary flakes of different sizes.

The assemblage of retouched artefacts comprises mainly scrapers (Table S10, Fig. 4 no. 11, 12) and notched tools whereas bifacial tools are mostly made on flakes or chunks, and documented by three exhausted examples (Fig. 4 no. 1–3), a fragment of a leaf-shape point, and by few preforms (Fig. 4 no. 4, 5, 8). In some flakes, the platform is lipped, a feature that is generally associated with the use of a soft hammer and bifacial shaping. In the collection, it is worth noting the presence of a scraper on a cortical blank with a Quina scaled retouch and one *groszak*. Although the lithic assemblage of Stajnia Cave is highly fragmented and bifacial backed knives (*Keilmesser*) are absent, the presence of bifacial tools, leaf point and *groszak* supports the association with the Central European Micoquian.

## Discussion

The combination of chronological, paleogenetic, morphometric and archaeological analyses from Stajnia Cave reveals new insights into Neanderthals in Micoquian cultural contexts. The mtDNA analysis of the molar S5000 indicates that the sample belongs to the Neanderthal mtDNA clade that separated from the Altai (Denisova 5), Denisova 15 and Scladina mitochondrial genomes ~ 170 ka (95% HPDI: ~ 138–203 ka). Based on the maximum parsimony results, the mtDNA sequence of S5000 has fewest differences to Mezmaiskaya 1 Neanderthal and shows a more distant relationship to the later Western European Neanderthals, among which some, such as Mezmaiskaya 2 and Feldhofer, are also associated with the Micoquian industry (Fig. S2, S3).

The genetic date obtained through the molecular branch shortening of the tooth S5000 of Stajnia Cave is ~ 116 ka (95% HPDI: ~ 83–152 ka), making it older than the late Neanderthals dated to MIS 3 (Fig. 3). Furthermore, both the Bayesian tree and the Maximum Parsimony trees (Fig. 3, S2, S3) reveal a more distant relationship of Stajnia S5000 mtDNA to the contemporaneous Neanderthals of Scladina and Hohlenstein-Stadel dated to ~ 120 ka (95% HPDI:, ~ 82–161 ka, and 95% HPDI: ~ 69–187 ka, respectively; Table S8). In turn, the mtDNA of Stajnia S5000 is closest to the one of Mezmaiskaya 1 Neanderthal, with both of them branching off ~ 152 ka (95% HPDI: ~ 124–182 ka) from the mtDNA genomes of Okladnikov 2, Denisova 11 (DC1227), and the later European Neanderthals.

Although S5000 was found in layer D2, the genetic age reveals an older chronology for the specimen suggesting that post-depositional frost disturbances might have moved the tooth from its original location. However, since Eemian sediments (123–109 ka) are absent in the cave and no archaeological materials were found in unit G (MIS 5c, 96–87 ka) and F (MIS 5b, 87–82 ka), the most parsimonious explanation is that the tooth belongs to layer E2 dated to MIS 5a (82–71 ka). This would indicate that S5000 is the oldest Neanderthal fossil found thus far in Central-Eastern Europe.

The sub-stage MIS 5a marked an important behavioural shift for Neanderthals in Central and Eastern Europe that adapted to new ecological conditions and developed novel land-use strategies for coping with the increased migratory radius of cold adapted fauna. In order to ensure sufficient intake of calories in low biomass environments, Neanderthals supplemented their toolkits with a wide range of bifacial knives. These stone tools have been proven to be efficient and flexible in the context of high mobility since frequent re-sharpening could assure a long use life and, in the case of raw materials shortage, they could be turned into cores^39,40^. In Poland, during this interpleniglacial stage, the Central European Micoquian is well represented and, beyond the taphonomic issues, the lithic assemblage of Stajnia displays a set of diagnostic pieces that are common also in other key sites in the Kraków-Częstochowa Upland, Polish Carpathians, Moravia and Germany (Fig. 1, SI Section 1, 6). In a broader perspective, similar technical behaviours are also found between the Eastern Carpathians and the lower Volga and especially in Crimea and Northern Caucasus (SI Section 1, 6). A statistical comparison between techno-typological features of several sites from the Central European Micoquian, the Eastern Micoquian and Chagyrskaya Cave indicates strong similarities between the assemblages from the West, the Caucasus and the Altai^17^. The common and recurrent leitmotiv between these sites is the high fragmentation of the *chaînes opératoires*, and the import on-site of configured cores, flakes and retouched tools^14,16,17,41–43^. These characteristics are typical of high mobility patterns and recurrent short-term occupations^44^.

The corroboration of the increased mobility of Neanderthals from periglacial and boreal environments of Central-Eastern Europe at the onset of the Micoquian is also confirmed by other genetic studies. Recently, comparisons of Neanderthal nuclear genomes suggest a population turnover from Western Europe to the Altai region after 90 ka^1^ whereas the continuous gene flow between Neanderthals and Denisovan during the last 100 ka, and the almost complete absence of Denisovan ancestry in European Neanderthals suggests recurrent dispersals eastwards to Altai^45^. Furthermore, a long distance migration of Neanderthals carrying a Micoquian toolkit has been discovered at Chagyrskaya Cave in Altai^17,18^ whereas movements of Micoquian groups (*Keilmessergruppen*) from Central Europe to other western regions have also been documented in the Netherlands, Belgium, and France^46–48^. These evidences suggest that the foraging radius of Neanderthals living in steppe/taiga environments could have been bigger than previously envisaged. Thus, the high mobility patterns on a west–east axis across the Northern and Eastern European Plains could explain the widespread distribution and prolonged production of specific Micoquian stone tools in comparison with the other Middle Palaeolithic facies. Although some authors propose the existence of chronological trends^13^, the “core” of the Micoquian technical behaviours remained stable over time. The variability recorded in some sites is generally related to the dimension and shape of bifacial knives^13^, or different frequencies of retouched artefacts^49^. However, these differences could have been generated by the dimension of raw material nodules, the recurrent re-sharpening of the bifacial cutting-edges, or to the site function rather than being the result of variations in knapping concepts. This technological stability also renders difficult the chronological attribution of Micoquian finds without the support of a precise age determination.

From the archaeological perspective, no technological breaks are documented in Micoquian^13,21^. However, Stajnia S5000 and Mezmaiskaya1, two oldest Neanderthal specimens associated with Micoquian, also have the smallest number of differences between their mtDNA genomes, and fall outside of the mtDNA variation observed in late Neanderthals found in Micoquian contexts (Fig. 3, S2, S3). Furthermore, data on nuclear DNA reveals that Mezmaiskaya 2 has more affinities with Western Neanderthals than with an earlier group from the same region^19^. From an archaeological point of view, the glacial climatic conditions of MIS 4 (71–57 ka) might have caused a severe decline of Neanderthal demography in Western Europe and the Caucasus, which is documented in the substantial reduction in the number of Neanderthal archaeological sites. Archaeological sites dated to the beginning or the final MIS 4 are scarce and mostly located south of 45° N latitude^50^. Although low-density Micoquian occupations are found at Geißenklösterle Cave^51^, and Garzweiler open-air sites (Germany)^52^, in general, a demographic gap is documented during the Pleniglacial across the North and the Eastern European Plains. A similar situation is recorded in Crimea where, even if the environment was characterised by boreal habitat, the sole Micoquian evidence is found at Kabazi II^53^. From this perspective, since the Micoquian techno-complex persisted in Central-Eastern Europe during MIS 3 without significant technological variations, and it is absent in the Mediterranean regions (Iberian Peninsula, Italy and the Balkans), the most parsimonious explanation is that the southern Micoquian fringes (eastern France and/or Hungary) could have been the sink areas that contributed to the repopulation of Central Europe during the climatic amelioration. Then, after the recolonization of the northern territories, other Neanderthal groups dispersed back into the Caucasus moving south across the Prut and Dniester basins. Although radiometric and genomic data are still missing from the Micoquian evidences in eastern France and Hungary, the genetic similarity between Mezmaiskaya 2 and the late western Neanderthals, and the absence of bifacial backed knives in the purported glacial refugia is striking and reduce the possible explanations.

## Conclusion

The current archaeological and paleogenetic evidences from the Late Middle Palaeolithic in Central Europe and the Caucasus portray a complex scenario of Neanderthal dispersal, local extinction and repopulation from the western regions. The multi-disciplinary investigation on the archaeological record of Stajnia Cave confirms the foraging activities in Kraków–Częstochowa Upland during MIS 5a and spotlights the affinity between Neanderthals from Southern Poland and the Caucasus. The technological similarities between the Central European Micoquian and the Eastern Micoquian are likely the result of increasing mobility of Neanderthal groups that frequently moved across the Northern and Eastern European Plains chasing cold adapted migratory animals. In this scenario, Poland, with its position at the crossroad between the Western and Eastern European Plain is a crucial area for disclosing the patterns of Neanderthal dispersal from the West to Central Asia. Further studies on the other teeth from Stajnia Cave will be pivotal for disentangling the timing of these movements and the social relation with the other Micoquian Neanderthals.

## Materials and methods

### Radiocarbon dates

The collagen for the 5 animal bones and the S5000 tooth were extracted at the Department of Human Evolution, Max Planck Institute for Evolutionary Anthropology (MPI-EVA) in Leipzig (Germany) following the pretreatment procedures in Talamo and Richards^54^ and Fewlass, et al.^55^ respectively.

### Radiocarbon on the S5000 tooth

Due to the limited material available, we extracted collagen from a very small sample of the S5000 tooth using the method described in^55^. Briefly, 70.5 mg dentine was removed with a dentistry drill and demineralised in HCl 0.5 M (4 °C) for one day. The sample was washed with MilliQ water and treated with NaOH 0.1 M to remove humic acid contamination (10 min) and re-acidifed with 0.5 M HCl. The sample was gelatinised in acidic water (pH3) at 70 °C until solubilised. The gelatin was then filtered to remove > 80 µm particles (Ezee filter, Elkay labs, UK) and ultrafiltered to separate the high molecular weight fraction (Sartorius VivaSpin Turbo 15–30 kDa molecular weight cut off (MWCO)). Ultrafilters were pre-cleaned according to^56^. After freeze-drying, ~ 0.5 mg collagen was weighed into a tin cup and measured in a ThermoFinnigan Flash elemental analyser (EA) coupled to a Thermo Delta plus XP isotope ratio mass spectrometer (IRMS). The extracted collagen (2.8 mg) was then weighed into a pre-cleaned tin cup and sent to the Curt-Engelhorn-Centre for Archaeometry Klaus-Tschira-AMS facility in Mannheim, Germany (MAMS) where it was combusted, catalytically converted to graphite and measured in the MICADAS-AMS^57^. Two small aliquots of a background bone (> 50,000 BP) were pretreated and measured alongside the S5000 tooth to monitor lab-based contamination. The age calculation was performed in BATS^58^ using measurements of the background collagen samples and standards measured in the same magazine, with an added external error of 1‰ (as per standard practice). The collagen yield (5.4%) from S5000 was sufficient for analysis with EA-IRMS and AMS dating. The ^14^C age AMS result was 22,480 ± 70 BP. This result is most improbable for a Neanderthal specimen, and we consider it to be an underestimation of the true age of the sample, probably due to contamination of the sample with glue applied post-excavation.

#### Radiocarbon on animal bones

Collagen from five animal bones was extracted at the Department of Human Evolution, Max Planck Institute for Evolutionary Anthropology (MPI-EVA) in Leipzig (Germany) following the pretreatment procedures in Talamo and Richards^54^ (MPI-Code: S-EVA). The outer surface of the bone sample is first cleaned by a shot blaster and then 500 mg of the whole bone is taken. The samples are then decalcified in 0.5 M HCl at room temperature until no CO_2_ effervescence is observed. 0.1 M NaOH is added for 30 min to remove humics. The NaOH step is followed by a final 0.5 M HCl step for 15 min. The resulting solid is gelatinised following Longin^60^ at pH 3 in a heater block at 75 °C for 20 h. The gelatine is then filtered in an Eeze-Filter (Elkay Laboratory Products (UK) Ltd.) to remove small (> 80 µm) particles. The gelatine is then ultrafiltered^61^ with Sartorius “VivaspinTurbo” ultrafilters (30 kDa MWCO). Prior to use, the filter is cleaned to remove carbon containing humectants^62^. The samples are lyophilised for 48 h. C:N atomic ratios, and collagen yields were measured to determine the extent of collagen preservation. Bones with > 1% weight collagen and C:N ratios in the range 2.9–3.6 pass the evaluation criteria for collagen to proceed with the AMS analysis^59^. Samples were graphitised and dated by AMS at the Mannheim facility (laboratory code MAMS;^57^).

The resulting date was corrected for a residual preparation background estimated from pretreated ^14^C-free bone samples, kindly provided by the Oxford Radiocarbon Accelerator Unit (ORAU). All the samples pretreated at the MPI-EVA passed the evaluation criteria for good quality collagen and are reported in Table S1.

### Tooth morphology

High-resolution µCT images of the tooth S5000 (Fig. 2), an upper right second molar (RM2), were obtained with an X-ray micro-computed tomography (XMT) scanner (GE Sensing & Inspection Technologies, phoenixjx-ray, Wunstorf, Germany) using the following scan parameters: 100 kV, 70 mA, with a 0.1 mm copper filter, and isometric voxels of 7.49 microns3. Volume data were reconstructed using isometric voxels of 30 µm. The image stacks were segmented in Avizo 9.1 (Thermo Fisher Scientific), to separate the enamel from the dentine and to reconstruct 3D digital models of the tooth, which were refined (i.e. cleaning processes and corrections of defects to create fully closed surfaces) in Geomagic Design X software (3D Systems Software).

### S5000 mtDNA extraction and library preparation

Seven samples of between 8.8 and 41 mg of tooth powder (Table S3) were removed from the S5000 specimen using a sterile dentistry drill. DNA was extracted using the DNA extraction method of^63^ with modifications described in^64^, either manually or on an automated liquid handling platform^65^. In an attempt to remove present-day human and microbial DNA contamination from the specimen, one of the tooth powder aliquots was treated with 0.5 M sodium phosphate buffer, followed by 0.5% hypochlorite solution^64^, before DNA extraction. Aliquots of each extract, as well as phosphate buffers, were converted into single-stranded DNA libraries^64,66,67^ yielding between 4.31 × 10^8^ and 4.09 × 10^9^ library molecules (Table S3). The libraries were then amplified^68^ and tagged with two sample-specific sequences^69^. Amplified libraries were enriched for hominin mitochondrial DNA (mtDNA) using a hybridisation capture method described elsewhere^70^ and modern human mtDNA as a bait. Enriched libraries were pooled together with libraries from other experiments and sequenced on the Illumina MiSeq and HiSeq 2,500 platforms in paired-end mode^69^. Base calling was carried out using Bustard (Illumina) for MiSeq runs and FreeIbis^71^ for HiSeq runs. After merging overlapping paired-end reads to reconstruct full-length molecule sequences^72^ and mapping to the revised Cambridge reference sequence (rCRS, NC_0120920) using BWA^73^ with ‘ancient’ parameters^74^, only sequences that were at least 35 bp long and had a mapping quality greater or equal to 25 were retained for subsequent analyses. After removing duplicate sequences with identical alignment start and end points (https://bitbucket.org/ustenzel/biohazard-tools), the number of sequences originating from unique mitochondrial DNA fragments ranged from 4,964 to 78,191 (Table S3).

### Vertebrate analyses

Each vertebrate fossil specimen was identified to the taxonomic and anatomical level on the base of comparisons with the mammal collections of the Department of Palaeozoology of the Institute of Environmental Biology at the Wrocław University, and the Institute of Systematics and Evolution of Animals at the Polish Academy of Sciences – Krakow. Bone quantification comprises the number of identifiable specimens (NISP) if possible to species level, and otherwise grouped per size class, and minimum numbers of individuals (MNI) calculated after Lyman^75^. Taphonomic investigations have to date, only been conducted on the bones used in the radiocarbon dating program. Recorded features include bone surface weathering (stages after Behrensmeyer^76^), primary and secondary breakage patterns, evidence of sediment, water, and chemical abrasion such as striations, smoothing, and root etching, as well as a carnivore (gnawmarks, pits and punctures), rodent (toothmarks), and human (cutmarks, impact fractures, chopmarks, and bone working) modifications.

### Lithic analysis

The lithic assemblage is under study and, in this examination, we report data on the items bigger than 2 cm. The analysis is carried out following the *chaîne opératoire* approach^77,78^. The Levallois and discoid technology is identified following the criteria defined by Boëda^78^. The intermediate core morphologies, characterised by a hierarchisation of the flaking surfaces and core configuration with secant fracture planes, are considered hierarchised, and discriminated on the base of the direction of detachments (e.g. unidirectional, bidirectional or centripetal) as described in Picin^79^, Picin et al.^80^. The study of the flake assemblage is performed by analysing the presence (cortex > 50% = cortical flake; cortex < 50% = semi-cortical flake) or absence of cortex, the number and direction of detachments on the dorsal face, the angle and the type of striking platform, the flaking axis, the presence of knapping accidents (e.g. overshot and hinged removal, siret fracture), and the retouch. Retouched tools are distinguished following Bordes^81^ typological list whereas denticulates and notched tools are analysed according to Picin, et al.^82^. Bifacial tools are described following the typological list of Bosinski^83^ and the technological approaches illustrated by Boëda^84^.

## Supplementary information

Supplementary file1.

## Data Availability

The Stajnia S5000 mitochondrial consensus sequence reported in this paper is available in GenBank under the accession code MT795654. The aligned mitochondrial DNA sequences are deposited in the European Nucleotide Archive under the accession number PRJEB39529.
