# Author Correction: New perspectives on Neanderthal dispersal and turnover from Stajnia Cave (Poland)

**DOI:** 10.1038/s41598-022-08141-z

**Published:** 2022-03-08

**Authors:** Andrea Picin, Mateja Hajdinjak, Wioletta Nowaczewska, Stefano Benazzi, Mikołaj Urbanowski, Adrian Marciszak, Helen Fewlass, Marjolein D. Bosch, Paweł Socha, Krzysztof Stefaniak, Marcin Żarski, Andrzej Wiśniewski, Jean‑Jacques Hublin, Adam Nadachowski, Sahra Talamo

**Affiliations:** 1 Department of Human Evolution, Max Planck Institute for Evolutionary Anthropology, Deutscher Platz 6, 04103 Leipzig, Germany; 2 Department of Evolutionary Genetics, Max Planck Institute for Evolutionary Anthropology, Deutscher Platz 6, 04103 Leipzig, Germany; 3 Department of Human Biology, Wrocław University, ul. Kuźnicza 35, 50‑138 Wrocław, Poland; 4 Department of Cultural Heritage, University of Bologna, Via degli Ariani 1, 48121 Ravenna, Italy; 5 Independent Researcher, Warsaw, Poland; 6 Department of Palaeozoology, Institute of Environmental Biology, Wrocław University, Sienkiewicza 21, 50‑335 Wrocław, Poland; 7 McDonald Institute for Archaeological Research, University of Cambridge, Downing Street, Cambridge, CB2 3ER UK; 8 Division of Palaeozoology, Department of Evolutionary Biology and Ecology, Wrocław University, ul. Sienkiewicza 21, 50‑335 Wrocław, Poland; 9 Polish Geological Institute, National Research Institute, Rakowiecka 4, 00975 Warsaw, Poland; 10 Institute of Archaeology, University of Wrocław, Szewska 48, 50‑139 Wrocław, Poland; 11 Collège de France, 11 Place Marcellin Berthelot, 75005 Paris, France; 12 Institute of Systematics and Evolution of Animals, Polish Academy of Sciences, Sławkowska 17, 31‑016 Kraków, Poland; 13 Department of Chemistry G. Ciamician, University of Bologna, Via Selmi 2, 40126 Bologna, Italy

Correction to: *Scientific Reports* 10.1038/s41598-020-71504-x, published online 08 September 2020

Marjolein D. Bosch was omitted from the author list in the original version of this Article.

The Author contributions section now reads:

“W.N., A.N. and S.T. designed research; A.P., M.H., W.N., S.B., M.U., A.M., H.F., M.D.B., P.S., K.S., M.Ż., A.W., A.N. and S.T. performed research; A.P., M.H., W.N., S.B., M.U., A.M., H.F., M.D.B., P.S., K.S., M.Ż., A.W., A.N. and S.T. analysed data; A.P., M.H., S.T., W.N. and S.B. wrote the paper with the collaboration of all the co-authors.”

The original Article and its accompanying Supplementary Information file have been corrected.

Mikolaj Urbanowski disagrees with this Author Correction.

