# Supplementary material for: New perspectives on Neanderthal dispersal and turnover from Stajnia Cave (Poland)
Source: Sci Rep. 2020 Sep 8;10:14778. doi: 10.1038/s41598-020-71504-x (PMC7479612; doi:10.1038/s41598-020-71504-x)
Supplement: Supplementary file 1 — Supplementary file1. [file 41598_2020_71504_MOESM1_ESM.docx]

**Supplementary Information for**

**New perspectives on Neanderthal dispersal and turnover from Stajnia Cave (Poland)**

Andrea Picin, Mateja Hajdinjak, Wioletta Nowaczewska, Stefano Benazzi, Mikołaj Urbanowski, Adrian Marciszak, Helen Fewlass, Marjolein D. Bosch, Paweł Socha, Krzysztof Stefaniak, Marcin Żarski, Andrzej Wiśniewski, Jean-Jacques Hublin, Adam Nadachowski, Sahra Talamo

Corresponding authors: Andrea Picin

Email: [andrea_picin@eva.mpg.de](mailto:andrea_picin@eva.mpg.de)

**This PDF file includes:**

Supplementary text

Figures S1 to S4

Tables S1 to S11

SI References

**Section S1: The Central European and Eastern Micoquian**

After the Eemian (MIS 5e), the European environment during the Late Middle Palaeolithic (MIS 5d – MIS 3) was characterized by frequent climatic oscillations ^1,2^. In Central and Eastern Europe, these climatic conditions favoured the development of steppe/taiga vegetation ^1^ and the migration of cold adapted fauna from the Arctic (e.g. woolly rhino, woolly mammoth, reindeer) ^3^. Thus, after a long period of behavioural adaptation to forested habitats and semi-sedentary faunal communities ^4^, Neanderthals coped with new ecological conditions characterized by extreme seasonality and reduced distribution of resources with decreasing temperatures ^5,6^. In response to these new environmental conditions, Neanderthals of Central and Eastern Europe modified their technical behaviours and, in addition to the commonly prepared core technologies, started to produce a wide range of asymmetric bifacial tools, leaf-shaped artefacts and bifacial scrapers ^7,8^. This techno-complex is generally known as Micoquian, due to the similarities with the bifacial tools of the French site La Micoque ^9^, or *Keilmessergruppen*, stemming from the German term for an asymmetric bifacial knife, the *Keilmesser* ^10,11^. Since the production of these bifacial knives spread between eastern France ^12-14^ and the northern Caucasus ^15,16^, the examples recorded between the Eastern Carpathians and the lower Volga are generally called Eastern Micoquian whereas the other examples are named Central European Micoquian ^8,17-19^.

The Central European Micoquian is well documented during the interpleniglacial periods (MIS 5c, MIS 5a and MIS 3) in Poland, Germany, Czech Republic, and Slovakia ^7,17,20-23^ with fringes in Hungary ^24^ and north-eastern France ^25-27^. Although some authors support a long chronology rooted in the late Acheulean ^17,28^, the Micoquian developed across the North European Plains only after the Eemian ^7,29^. Generally, the Micoquian lithic industry is characterized by discoid, hierarchized or Levallois core technology, a high number of scrapers, small circular scrapers (called *groszaks*), bifacial tools and asymmetric bifacial backed knives ^30^. These latter tools were produced using the plano-convex reduction strategy ^31^, and in the archaeological record artefacts with different shapes and sizes are documented ^9,32-36^. Typologically, these stone tools are discriminated following the morphologies found at some key sites, and divided in types Bockstein, Balver or Buhlener *Keilmesser*, *Prądnik* or *Klausennische* *Messer*, Lichtenberger *Keilmesser*, and Königsaue (or Wolgograd) *Keilmesser* ^7,9^. A distinctive feature of the Central European Micoquian of southern Poland, Germany and eastern France is the use of the *prądnik* technique (also called lateral tranchet blow), a technical procedure for re-sharpening the distal end of bifaces and scrapers by detaching one or more spalls along the working edge ^14,36-38^.

In the Eastern Micoquian, the production of bifacial knives arose during MIS 5c/MIS 5a in Crimea at Kabazi II ^39^ and persisted up to the end of the Middle Palaeolithic from the northern Caucasus to the Urals, and from the Outer Eastern Carpathians to the Volga basin ^8,15,40-43^. The technological features of the lithic industries of the Eastern Micoquian are very similar to those recorded in Central Europe. Discoid, hierarchized or Levallois core technologies were used for the production of flakes that were successively shaped in side-scrapers and convergent tools ^41,44^. Asymmetric backed knives (*Keilmesser*), groszaks and the use of the *prądnik* technique are less frequent or absent in the Eastern regions whereas symmetric bifaces and leaf-shaped artefacts are widespread ^16,19,45,46^. In Crimea, the distinction between different frequencies of stone tools permitted the recognition of different facies: a) the Ak-Kaya facies shows the highest percentages of bifacial tools, simple scrapers, and large tools and the lowest percentage of unifacial convergent tools; b) the Kiik-Koba facies has the lowest percentage of bifacial tools, simple scrapers, and the highest percentage of convergent tools; c) the Staroselian facies falls intermediate between the other two for all of these attributes ^44^.

In Central and Eastern Europe, the Neanderthals’ technical behaviour was varied and several lithic assemblages without the production of bifacial knives are also documented, and attributed to the facies Levallois-Mousterian ^17,20,45,47^. Current hypotheses on the interpretation of these techno-complexes suggest either the presence of two different cultural traditions ^17,44^ or the outcome of different mobility patterns carried out by the same groups of Neanderthals during their annual land-use cycles ^30,48,49^. Beyond these different interpretations, echoing the cultural *vs* functional debate of the 1970s ^50,51^, the marked separation between the two facies is often prompted by greater attention to bifacial tools over the core reduction strategies sustaining their primary roles in defining regional patterns ^7,16,52,53^. Thus, the technological features of the Levallois-Mousterian could be comprised in the broad technological plasticity of the Micoquian of the Central and Eastern Europe. Recently, the reassessment of the lithic assemblages of Königsaue open-air site (Germany), the sole example of interstratification between *Keilmessergruppen* and Levallois-Mousterian in Central Europe, evidenced a common technological background between the two facies and pointed out that the main difference is recorded in the transport of the artefacts off-site rather than being two discrete cultural traditions ^54^. Another example is documented at Hallera Avenue open-air site in Wrocław (Poland) where a few bifacial shaping by-products and tools are found in a Levallois-Mousterian context ^55,56^. Conversely, in Crimea, a strict separation between the two facies is favoured and the appearance of Micoquian elements in the Levallois-Mousterian assemblages at Kabazi V rock-shelter is interpreted to be the result of mechanical mixture ^57^. Beyond these techno-typological differences, Neanderthals from the North and Eastern European Plains share similar behaviours in land use with patterns of high mobility across the territory ^21,23,54,58-60^. These comportments do not change through time and are maintained in both periglacial and boreal environments ^21,54,59^.

**Section 2: Stratigraphic sequence of Stajnia Cave**

The stratigraphic sequence of Stajnia Cave is mainly characterized by cave loams consisting of limestone rubble, sand, silt and clay (Fig. 1D) ^61^. Post-depositional frost disturbances, partial sediment sinking, and modern distortions make the stratigraphy complicated. At the base is unit G, composed of orange-brown sandy cave loam with a small amount of limestone rubble, overlaid by unit F that consists of light-grey to brown sandy cave loams with low admixture of limestone rubble. Above unit F lays unit E divided into layer E2, consisting of light-grey sandy-silty cave loams with significant limestone rubble and containing few Middle Palaeolithic lithic artefacts, and layer E1, archaeologically sterile and composed of a large amount of limestone rubble. Above layer E1, unit D is the part of the sequence richest in Middle Palaeolithic artefacts and where the Neanderthal teeth were found. Unit D is divided into four layers (D3, D2, D2b, and D1considering an additional layer D1a for layer D1 sediments found in secondary context in some part of the cave). Layer D3 consists of ~30 cm of dark brown sandy-silty-clayey cave loam with an admixture of fine limestone rubble. Layer D2 is a light brown cave loam with rubble whereas layer D2b is a discontinuous structure, probably formed during the deposition of the sediments of layer D2, composed of light brown sandy-clayey-silty cave loam with limestone rubble. Layer D1 is ~50 cm of light brown cave loam with rubble. In trench 2, a portion of the layer is distinguished as D1b. The uppermost part of the sequence includes unit C (layers C6, C7, C18 and C19), in which the sediments are partly disturbed by cryoturbation, unit B (layers B0-B9) composed of some lenses of yellow sandy cave loam, and unit A, a grey-black humus with limestone rubble deposited during the Holocene and mostly removed during late Medieval times ^61^.

The chronological assessment of the sequence was carried out crossing dating methods with the geological features of the different units. Unit G was accumulated in a warmer climate and probably correlated with MIS 5c, whereas the sediments of unit F are related to slightly cooler conditions and are associated, most likely, to MIS 5b ^61^. The accumulation of sandy-silty loam in layer E2 suggests warm climatic conditions, possibly connected with MIS 5a. The sediment of layer E1 suggests a cold and harsh environment and is associated with MIS 4. A radiocarbon date on an ungulate bone from this layer is >49,000 years (Table S1). The sediments of unit D were accumulated during MIS 3. In layer D3, two fragmented bones of a bear and an ungulate are >49,000 years (Table S1). In layer D2b, the dating of two mammoth tooth fragments by U-Th series yielded ages of ~52,900 BP, and a radiocarbon date on an ungulate bone is >49,000 years (Table S1). In layer D1, an Optically Stimulated Luminescence (OSL) date of cave loam is 45,900 BP (GdTL-1127) whereas the radiocarbon dating of a mammoth tusk fragment gave a result of 44,600 ± 2,100 ^14^C BP (OxA-24944), and of an ungulate bone yielded an age of 44,590 ± 690 ^14^C BP (Table S1). Another radiocarbon date on a bear tooth, found in layer D1, gave an age of >49,000 years (Poz-28892). In layer B, the results of 13,500 ± 60 ^14^C BP (Poz-28891) on a maxilla of a saiga by the radiocarbon method ^62^ and of 8,950 years (GdTL-1126) on the cave loam by the OSL method, indicate an association with MIS 2 ^61^.

**Section 3: tooth S5000**

**Morphological description of the tooth S5000**

The S5000 specimen discovered in Stajnia Cave was described by ^63^ as a permanent maxillary right second molar (RM^2^). The crown is worn (wear stage 3 according to Molnar ^64^), and post-mortem events caused enamel loss in the lingual side near the cervix. The lingual root is well preserved (i.e., where DNA sampling was undertaken), but both buccal roots were damaged by post-mortem events. Despite being fairly worn, four main cusps can still be recognized on the occlusal surface (^63^: Fig. 1 p. 412): the paracone (Pa), metacone (Me), hypocone (Hy) and protocone (Pr).

Specimen S5000 was diagnosed by ^63^ as belonging to Neanderthal based on the presence of a combination of traits frequently observed in Neanderthal M^2^s, such as: relatively large hypocone, specific pattern of the relative area of cusp base (Pr> Pa> Hy> Me) ^65-67^ and the presence and form of the subvertical grooves on the interproximal facets (distal and mesial) of the crown ^68^.

The buccolingual (BL = 11.0 mm) and mesiodistal diameter (MD = 10.72 mm - value corrected for interproximal wear; 9.6 mm - uncorrected value), (^63^: ESM 4.3) and the values of two indices (the Robustness Index (CBA = BL x MD) and the Crown Shape Index (CSI = BL/MD x 100)) of the S5000 specimen are presented in Table S2, along with data about crown metric traits of M^2^ hominin samples collected from the scientific literature as well as the number of standard deviations (Z-scores ) of the S5000 tooth from the M^2^ hominin sample means. The results indicate that the S5000 BL diameter is nearest to the mean of recent *H*. *sapiens* (RHs) but it does not depart from the mean established for Early Neanderthals (EN), Late Neanderthals (LN), Skhul/Qafzeh early *H*. *sapiens* (SQ) and Late Upper Palaeolithic *H*. *sapiens* (LUPHs) (Table S2). With regard to the MD diameter, specimen S5000 is nearest the mean values established for SQ, EN, LN and EUPHs (Table S2). The Z – scores computed for the two indices, i.e. CBA and CSI, of S5000 are nearest the means established for Upper Palaeolithic *H*. *sapiens* and Neanderthals (for CBA), and Early *H*. *sapiens* and Neanderthals (for CSI) (Table S2). The results presented above do not exhibit significant taxonomic indications and are congruent to Bailey ^65,^Bailey ^66^ observation that crown diameters provide limited information for the taxonomic assessments of hominin M^2^s. However, the bivariate analysis of MD and BL crown diameters was carried out using equiprobable ellipses (see e.g. ^69^) in PAST software ^70^ and in the obtained bivariate plot specimen S5000 falls outside the equiprobable ellipses (95%) of all hominin groups of our comparative sample, except Neanderthals (Fig. S1). Topographic information on the enamel-dentine junction (EDJ) surface (see main text and Fig. 2) supports a Neanderthal attribution of the tooth. The EDJ traits of the S5000 tooth were described according to the terminology and classification provided by the Arizona State University Dental Anthropology System (ASUDAS) ^71,72^ and compared to that observed in Neanderthal M^2^ s^72,73^.

**Section 4: Ancient DNA**

**Authentication of ancient DNA fragments in Stajnia S5000**

Ancient specimens are often contaminated by present-day human DNA ^74-76^, requiring the authentication of the hominin-like fragments retrieved from the Stajnia S5000 specimen. We, therefore, determined the frequency of C to T substitutions in the sequence alignments. These substitutions arise predominantly at the ends of ancient DNA molecules through deamination of cytosines (C) to uracils (U) ^77^, which are then read as thymines (T) by DNA polymerases, and are characteristic for authentic ancient DNA ^75,78^. Fragments with terminal C to T substitutions were observed at high frequency in all ten libraries, suggesting that at least some of the fragments were of ancient origin (Table S3), including those recovered from the phosphate buffer used for decontamination.

**Attributing the mitochondrial DNA of Stajnia S5000 to a hominin group and molecular DNA dating**

In order to determine whether the mtDNA of Stajnia S5000 originates from a modern human or a Neanderthal, we first studied the state of DNA fragments overlapping positions in the mitochondrial genome at which these two hominin groups differ ^79^. To alleviate the influence of substitutions derived from deamination, we ignored all forward strands where one of the possible states at an informative state was a C and all reverse strands where one of the possible states was G.

Given the present-day human DNA contamination estimates among all mtDNA sequences recovered from Stajnia S5000, we next reconstructed the mitochondrial genome sequence of Stajnia S5000 using deaminated fragments only and requiring a position to be covered by at least three sequences, of which two thirds had to be in agreement, and the base quality at the position was 20 or higher ^79^. In order to prevent deamination-induced C to T substitutions from affecting the reconstruction of the consensus, we converted Ts on the forward strands and As on the reverse strands in the first three and the last three positions of DNA fragments into Ns. After the realignment of the mtDNA sequences to the mitochondrial genome of Vindija 33.16 ^80^, five positions were still covered by two or fewer sequences and nine positions had less than two thirds of sequences supporting the same state. Thus, we exclude these positions from all comparative mtDNA genomes in our downstream analyses.

The multiple sequence alignment including Stajnia S5000 consensus sequence and the mitochondrial genomes of 54 present-day humans ^81^, ten ancient modern humans ^75,82-86^, 24 Neanderthals ^80,81,87-95^, four Denisovans ^96-99^, a hominin from Sima de los Huesos ^79^ and the chimpanzee ^100^ was generated using MAFFT ^101^.

We removed all the positions from the alignment containing missing data, which include positions we were unable to resolve in Stajnia S5000 mtDNA genome and the positions in other mtDNA genomes in the multiple sequence alignment which have missing data. We used jModelTest ^102^ to identify the best substitution model (TN93+I+G). The number of pairwise differences among the full mitochondrial genomes and after restricting the analyses to the coding region only (positions 577-16,023 of the rCRS) was calculated using MEGA7 ^103^ and the maximum parsimony trees (Fig S2 and S3) were generated using Parsimony ratchet from the R package *phangorn* ^104^.

We used Beast2 (version v2.4.7, ^105^) to estimate the molecular age of Stajnia S5000 mtDNA genome by taking advantage of ten Neanderthals and ten ancient modern humans (Table S6) that are directly radiocarbon dated as multiple calibration points ^82,106^. For this analysis we aligned the mitochondrial genome of Stajnia S5000 using MAFFT ^101^ to the mitochondrial genomes of 24 Neanderthals ^80,81,87-95^ , 54 present-day humans ^81^, ten radiocarbon dated ancient modern humans ^75,82-86^, and Denisova 3 mitochondrial genome ^97^ to be used as an outgroup. As before, all the positions containing missing data were removed from the alignment, and only the coding region of the mtDNA genome (positions 577-16,023 of the rCRS) was retained for the analysis ^82^.

The best fitting substitution model, as determined by jModelTest (version 2.1.7 ^102^, was Tamura-Nei (TN93) with a fraction of invariable sites and gamma distributed rate among the sites (TN93+I+G), as above. We investigated an uncorrelated log-normal distributed relaxed clock and a strict clock as two different models of rate variation, and a constant size and Bayesian Skyline ­as tree priors ^83^. We used the fixed mutation rate of 1.57x10^-8^ substitutions per site per year for the coding region ^82^. We set the date of present-day human mtDNA genomes to zero, and used uniform priors for ancient modern humans and Neanderthals that were directly dated (Table S6). For the undated Neanderthals, to estimate their age, we used a prior of the age ranging from 30,000 to 200,000 years ago with an initial value of 50,000 years ^89^. For each model, we carried out two independent Markov Chain Monte Carlo (MCMC) runs with 30,000,000 iterations each, sampling every 1,000 steps, and using 10% of the iterations as a burn-in. The four models were then compared to each other using the stepping stone and path sampling ^107^. We determined that the strict clock and Bayesian skyline tree prior best fit the data. We then used these model combinations and ran three independent MCMC runs with 75,000,000 iterations each, sampling trees and parameter values every 2,000 iterations. We combined the results of these runs with LogCombiner ^105^ and used 10% of the iterations as burn-in. The resulting Beast2 tree was visualised using FigTree (version: v1.4.2) (<http://tree.bio.ed.ac.uk/software/figtree/>).

**Section 5: The faunal assemblages**

The faunal remains found in Stajnia Cave include more than 13,500 bones, bone fragments and teeth identified as belonging to larger mammals and birds, and more than 31,000 determined teeth and bones of other vertebrates (including small mammals, reptiles and amphibians). The larger faunal remains were obtained directly during the excavations at Stajnia Cave and the smaller bone remains during the process of flotation, or sieving the sediments through sieves with mesh sizes of 3-4 mm. Most of larger mammals were identified to the species level, however a detailed zooarchaeological study of the total faunal assemblage is currently being conducted. Bone quantification comprises the number of identifiable specimens (NISP), if possible to species level, and otherwise grouped per size class, and minimum numbers of individuals (MNI) calculated after Lyman ^108^. The largest bones found during excavation and wet sieving were recorded, similarly to the other finds, within the stratigraphic unit and square meter, in layers five centimetres thick. However, in most cases the three-dimensional location was recorded of specific bones excavated *in situ*.

The fauna of larger mammals is composed of at least 26 species belonging to carnivores (Carnivora) and herbivores (Artiodactyla, Perissidactyla and Proboscidea) (total NISP = 4,698, total MNI = 604). Most identified large mammals were found in the archaeological levels of unit D (NISP = 1,310, MNI = 128) and older layers of unit C (MNI = 1,873, MNI = 204) (for the stratigraphic subdivision see *SI Appendix* Section 2 and ^61^, Fig. 9). Probably the deposition of at least part of the collection of larger mammal bones occurred as a result of human activity, but as already mentioned, taphonomic and archeozoological analysis has not yet been carried out.

The fauna of herbivorous mammals in Stajnia Cave is dominated by representatives of the so-called *Mammuthus-Coelodonta* Complex ^3,109,110^ and is represented by 9 species (Table S9). The reindeer (*Rangifer tarandus*) is the most abundant species found in all layers of this site, especially in archaeological units D and C. The material belonging to the reindeer are teeth, antlers and bone of the post-cranial parts of the skeleton. Based on the remains of antlers, it can be stated that the majority of individuals were represented by females ^111^. Steppe bison (*Bison priscus*) is also relatively common in the younger layers of unit C. Other species associated with the steppe and steppe-tundra habitat, namely the mammoth (*Mammuthus primigenius*), woolly rhinoceros (*Coelodonta antiquitatis*) and the wild horse (*Equus ferus*) were represented by single individuals. In the material of these species, there are only teeth or teeth fragments. The woolly rhinoceros is represented mainly by the remains of young individuals. Attention should be drawn to the presence of saiga antelope (*Saiga tatarica*), found only in unit B, a rare species in Poland ^62,112^. In upper layers of unit C (end of MIS 3), there were found a few remains of red deer (*Cervus elaphus*), which may indicate the presence of forests.

The carnivore paleocommunity from Stajnia Cave consists of 18 species (Table S9). The material is strongly fragmented, and there are almost no complete or large bones of large carnivores present, rather the material is dominated by isolated teeth and smaller bones. Contrary to these, in smaller carnivores long bones and mandibles dominate, especially in mustelids. Among three canids present, the most numerous is red fox (*Vulpes vulpes*), although also the wolf (*Canis lupus*) and Polar fox (*Vulpes lagopus*) are numerous. Amongst the wolf (*Canis lupus*) remains some specimens showed morphological similarity to steppe/cave wolf, an ectomorph typical for the Late Pleistocene open grasslands.

Bears are totally dominated by individuals of the last and most advanced member of cave bear speleoid lineage, *Ursus spelaeus ingressus*. Particularly high, especially in units D and C is also the number of young individuals, represented mostly by milk dentition. Among the most unique finds is the third phalanx of speleoid bear, from layer C18, one of the youngest ever found in Europe ^113^. The morphometric and morphological analysis confirmed the attribution of speleoid bear from Stajnia Cave to the *U*. *s*. *ingressus*. The typical individual of *U*. *s*. *ingressus* from Stajnia Cave was a very robust and large speleoid bear, with an average weight of 600-800 kg for males, and with an average weight of 250-400 kg for females. Multiple-coned and enlarged tooth cusps, broadened molar crowns and the lack of additional premolars confirmed their full herbivorous specialization. P4 morphotypes represented a multi-coned, broad type E, with enlarged main cusps. Some specimens from unit G hold many primitive features, and may partially represent an early stage of *spelaeus* lineage, transitional between *deningeri* and *spelaeus*. The brown bear (*Ursus arctos*) is much rarer, and in Late Pleistocene units E-C some material of *Ursus arctos priscus* was found, characteristic of open grasslands paleocommunities.

Mustelids are very abundant with the largest species including wolverine (*Gulo gulo*) and badger (*Meles meles*) represented by quite scare material. The presence of steppe polecat (*Mustela eversmanii*), a typical steppe species is noteworthy. An astonishing number of stoat (*Mustela erminea*) and weasel (*Mustela nivalis*) remains are mostly correlated with the activity of birds of prey. Remains of European pine marten (*Martes martes*) from Stajnia cave belonged mostly to moderately large and quite gracile individuals, typical of the warmer periods as well as postglacial and Holocene sediments. The obtained morphotypes of the lower dentition are also typical for modern *M*. *martes*. Some specimens of *M. martes* from units E, D and C are large in size and are correlated with cooler phases of the Late Pleistocene

The stoat (*Mustela erminea*) from Stajnia cave is characterized by robust build, subtly marked by a longitudinal bone furrow transversely along the lower dental series on the lingual side of the mandibular shaft, strongly marked extension of the posterior part of the P4 crown, a robust crown and a long M1 trigonid and long and narrow M1 talonid. The crown broadening on the base of the protoconid is especially well marked.

The most numerous material of carnivores is represented by weasel (*Mustela nivalis*), where males strongly outnumber females. The least common material belongs to small to medium and gracile individuals. The dominance of medium sized specimens can be correlated with a mild and rather warm climate. However, the presence of the small weasels might suggest that they lived in cooler periods with harsh weather and continental climatic influence.

Among cats, the European wildcat (*Felis silvestris*) is most numerous. Quite abundant is cave hyena (*Crocuta crocuta spelaea*), with the most numerous remains in unit C. The absence of coprolites and the discovery of single milk teeth suggest that in some layers the cave was not used as hyena den.

Taxonomic determination of birds, small mammals, and other remains (reptiles and amphibians) is not complete and therefore no comprehensive quantitative and spatial analysis of the fauna is currently available. The avifauna found in situ consists of almost 600 bone remains and is dominated by the genus *Lagopus* (*L*. *lagopus* and less frequently *L*. *muta*) as well as corvids, with dominant *Corvus monedula*, and the genus *Tetrao*. Many more bird remains, belonging to several smaller species, were found by wet screening in almost every sample. An extremely large number of remains of small mammals were obtained thanks to the flotation of almost all cave deposits. More than 25 species of small mammal remains were identified in all layers (ca. 50% of remains come from complex D). They include insectivorous species, first of all Soricomorpha, e.g. various species of shrews (genus *Sorex*) (NISP = 507) and bats (Chiroptera) (NISP = 377). Lagomorphs (Lagomorpha) include hares (*Lepus*) and pikas (*Ochotona*) (NISP=1,050). Rodents (Rodentia) distinctly prevail in the assemblage of Stajnia Cave and include over 31,000 items determined to the species level. In all layers of Stajnia Cave, small mammal remains represent “non-analogue” or “disharmonious” associations, typical for all sites from Kraków-Częstochowa Upland ^114-116^. The mix of steppe, boreal and woodland species is the most characteristic feature of such assemblages. The small mammal associations are dominated by inhabitants of well-drained or wet tundra or other open habitats, represented by the collared lemming (*Dicrostonyx torquatus*) (NISP = 5,400), Norway lemming (*Lemmus lemmus*) (NISP = 3,727), tundra vole (*Alexandromys oeconomus*) (NISP = 4,711), common and/or field vole (*Microtus arvalis/agrestis* group) (NISP = 3,101) and the most numerous European narrow-skulled vole (*Lasiopodomys anglicus*) (NISP = 13,036), previously known mostly under the name *Lasiopodomys gregalis* (in terms of changing the nomenclature, compare ^117^). These species constitute between approximately 91-94% of all remains of small mammals in all studied layers. In layers of units D and E the most numerous were *L. anglicus* (from 29 to 36%), *D. torquatus* (from 13 to 18%), *L. lemmus* (from 9 to 16%), *M.* *arvalis* / *agrestis* (from 15 to 20%) and *A.* *oeconomus* (from 12 to 15%). The next ecological group of taxa in the non-analogue assemblage belongs to inhabitants of steppe or other dry-open habitats, which are not very numerous in the small mammal assemblage of Stajnia Cave (frequency around 1-3%). Species such as the common hamster (*Cricetus cricetus*), grey dwarf hamster (*Cricetulus migratorius*) or steppe lemming (*Lagurus lagurus*) occur only in some layers. The third most numerous group comprise species living in diverse biotopes. In Stajnia Cave many of them are relatively moist and damp habitats. They typically inhabit densely-vegetated areas along the edges of lakes, streams and marshes which can be found in tundra, taiga and forest-steppe. Wet meadows, bogs, riverbanks and flooded shores are all important habitats. The most characteristic species belonging to this ecological group is the European water vole (*Arvicola amphibius*). The frequency of species inhabiting various kinds of woodland is low in all samples. However, vertebrate species found in all kinds of forests, densely-vegetated clearings, woodland edges and shrubs are present in almost every layer. Among small vertebrates, the most characteristic species of this kind is the bank vole (*Clethrionomys glareolus*), occurring with a frequency between approximately 2% and 4%. To this ecological group also belong the very rare Northern birch mouse (*Sicista betulina*), field mice (*Apodemus sylvaticus/flavicollis* group) as well as dormice (Gliridae) and squirrels (Sciuridae), present mainly in the upper layers. Although complete reconstructions of past ecosystems based on the fossil mammal cave assemblages are impossible (e.g., many species of small forested mammals can be underestimated), the correlation between climatic and ecological changes, and changes concerning the species constitution of the vertebrate fauna, is possible. The more temperate fauna at the bottom of the sequence (units G, F) was succeeded by colder fauna (units E, D, C), with a distinct predomination of tundra species towards the top of the sequence. The identified assemblages of rodents in layers from units D and E are characterized by very similar species composition and proportion of individual species. This situation may result from the accumulation of the described rodent assemblages in similar environmental conditions or in a relatively short time interval. However, these preliminary studies also indicate the presence of a mosaic of habitats, with forested and open areas, water streams and lakes, and swamps, as well as rocky areas, in all studied layers.

**Section 6: Stajnia Cave in the context of the Central European and Eastern Micoquian**

The preliminary study of the lithic assemblages of Stajnia Cave reveals a complex situation due to post-depositional processes that clearly displaced some artefacts between different levels. However, even if the assemblages are considered as a whole, the analysis indicates a high fragmentation of the *chaînes opératoires*, and the import *on-site* of configured cores, flakes and retouched tools (Table S10-S11). These features are typical of high mobility patterns and recurrent short-term occupations ^118,119^, a type of foraging behaviour common during the Middle Palaeolithic in Central Europe ^21,54,60,120^. The opening of the cave was probably too narrow for prolonged settlement, and the site could be considered as one of the logistical locations settled by Neanderthals during their forays in the Kraków-Częstochowa Upland.

Although the lithic assemblage of Stajnia Cave is highly fragmented and bifacial backed knives (*Keilmesser*) are absent, the presence of diagnostic pieces supports the association with the Central European Micoquian. Moreover, in Poland during MIS 5c-5a, the use of discoid and centripetal technology, and the import of few Levallois flakes is frequent in the lower levels of Wylotne rock-shelter ^121^, Biśnik Cave ^28^, Nietoperzowa Cave ^122^, Raj Cave ^123^, Piekary I ^124^ and at the open-air sites of Piekary II, Piekary III ^124^, and Zwoleń^125^. Conversely, in Ciemna Cave and Obłazowa Cave, cores are rare and the lithic assemblages of the lower and upper units are mostly characterized by flakes and retouched tools ^126,127^. Generally, secondary operative chains, such as hierarchized and simple unidirectional methods, are also recurrent among these sites, and their frequencies vary on the base of site function and occurrences of the occupations ^28,121,124,125^. During MIS 3, the composition of the lithic series remain unchanged with a dominance of discoid and centripetal (hierarchized and simple) technologies over Levallois, and the production of scrapers, bifacial tools, *prądnik* scrapers, and leaf-shaped artefacts ^23,121,126,127^.

In the Northern Caucasus, the early phase (MIS 5c - MIS 4) of the Micoquian techno-complex is mostly associated with the evidences from Mezmaiskaya layer 3-2B4, Ilskaya 1 and 2, and Hadjoh-2. At Mezmaiskaya Cave, the bulk of the lithic collection is composed of scrapers, retouched points (Mousterian point and Chokurcha point), small handaxes, leaf artefacts and bifacial backed knives, some of them similar to Central European type *Bocksteinmesser* ^16,128^. Few of these stone tools are made on high-quality Cretaceous flint from outcrops located 300 km north near the Azov sea ^58^. Cores are numerous in layer 3 (n°51) and 2B4 (n°48) but a detailed description is still missing, and they are attributed to the broad unidirectional single-platform category ^41^. At the open-air Hadjoh-2 layer 7, the site was used as a lithic workshop and include cores (n°55) exploited using the unifacial single-platform method, and, in few examples, the centripetal and polyhedral reduction ^129^. Within cortical and ordinary flakes, and two Levallois recurrent unidirectional blanks, several bifacial scrapers, handaxes and side-scrapers are documented. At open-air sites of Ilskaya 1, cores were reduced using the discoid and polyhedral methods whereas Levallois technology is rare ^130^. The flake assemblage includes cortical pieces, and by-products of the preparation and re-sharpening of bifacial and unifacial tools suggesting that the knapping events were carried out at the site. The collection of retouched tools is dominated by side-scrapers, small triangular handaxes, leaf points, and by several bifacial knives, typologically attributed to *Keilmesser*, *prądnik* tools and *Wolgogradmesser* (or Königsaue *messer*) ^128^. At the nearby location Ilskaya 2, the lithic collection is smaller and comprises several isolated artefacts such as scrapers, triangular points and few bifacial tools ^128^. At the onset of MIS 3, the main difference documented in the region is the decrease of the frequencies of bifacial backed knives and leaf-shaped artefacts, and an increase of the number of retouched points and bifacial scrapers ^41^. Beyond these typological differences, the character of the lithic assemblages remained Micoquian, as implied also by the presence of *prądnik* scrapers in the upper levels of Baranakha 4 ^131^ and Barakaevskaya Cave ^132^.

**Fig. S1:** Bivariate analysis of BL (buccolingual) and MD (mesiodistal) diameters of permanent upper second molars with 95% equiprobable ellipses of Atapuerca-Sima de los Huesos hominins (AT-SH, black ellipse, ∆ - as specimen), Neanderthals (NE, green ellipse, ○ - as specimen), early *Homo sapiens* (E Hs, purple ellipse, + as specimen), Upper Palaeolithic *Homo sapiens* (UP Hs, brown ellipse, ⅹ as specimen), the position of the Stajnia S5000 specimen was marked as blue point, two teeth of Denisovans were presented as pink ⸋. To conduct this analysis the raw data obtained from literature using in this study to calculate the two indexes (CBA and CSI) were used.


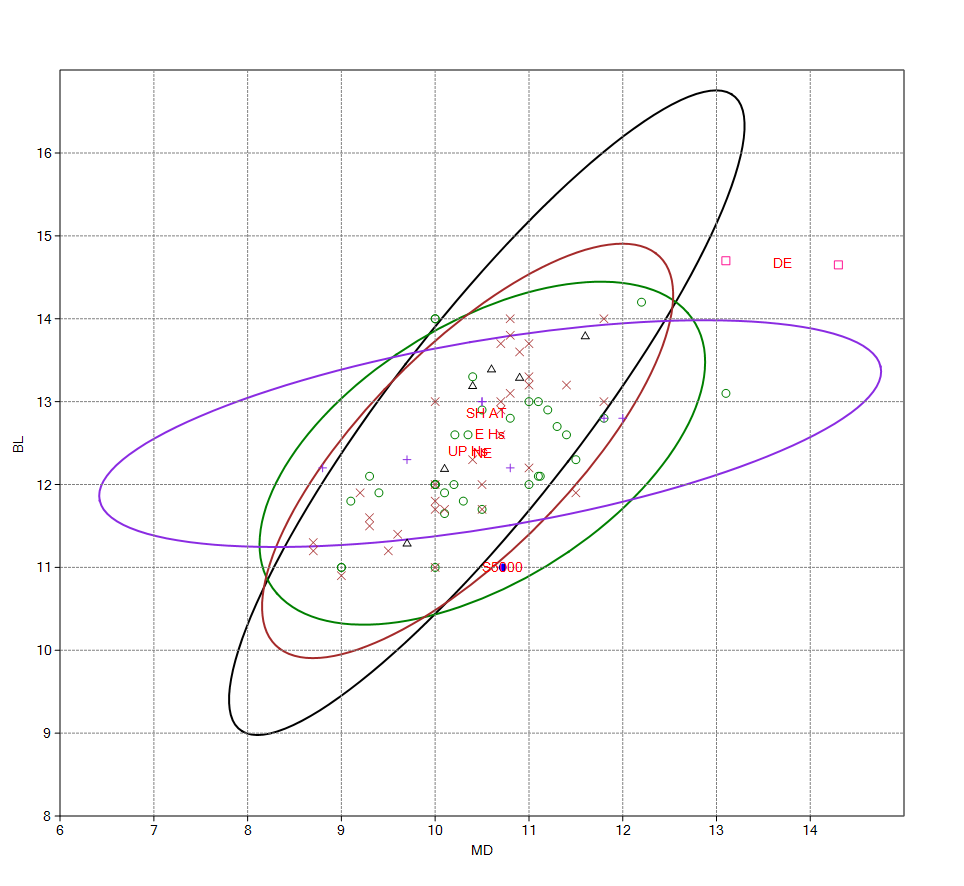


**Fig. S2**. Maximum parsimony tree relating the mitochondrial genome of Stajnia S5000 to the mitochondrial genomes of 24 Neanderthals, four Denisovans, 54 present-day humans, ten ancient modern humans, and one Sima de los Huesos individual. The mtDNA of Stajnia S5000 is indicated in red and the branches leading to the mtDNAs of present-day and ancient modern humans are collapsed for visualization purposes. The inferred number of substitutions per mtDNA sequence is given above each branch. A chimpanzee mtDNA was used to root the tree (not shown).





**Figure S3*:*** Maximum parsimony tree relating the mitochondrial genome of Stajnia S5000 to the mitochondrial genomes of 24 Neanderthals, four Denisovans, 54 present-day humans, ten ancient modern humans, and one Sima de los Huesos individual, restricted to the coding region. The mtDNA of Stajnia S5000 is indicated in red and the branches leading to the mtDNAs of present-day and ancient modern humans are collapsed for visualization purposes. The inferred number of substitutions per mtDNA sequence is given above each branch. A chimpanzee mtDNA was used to root the tree (not shown).

**
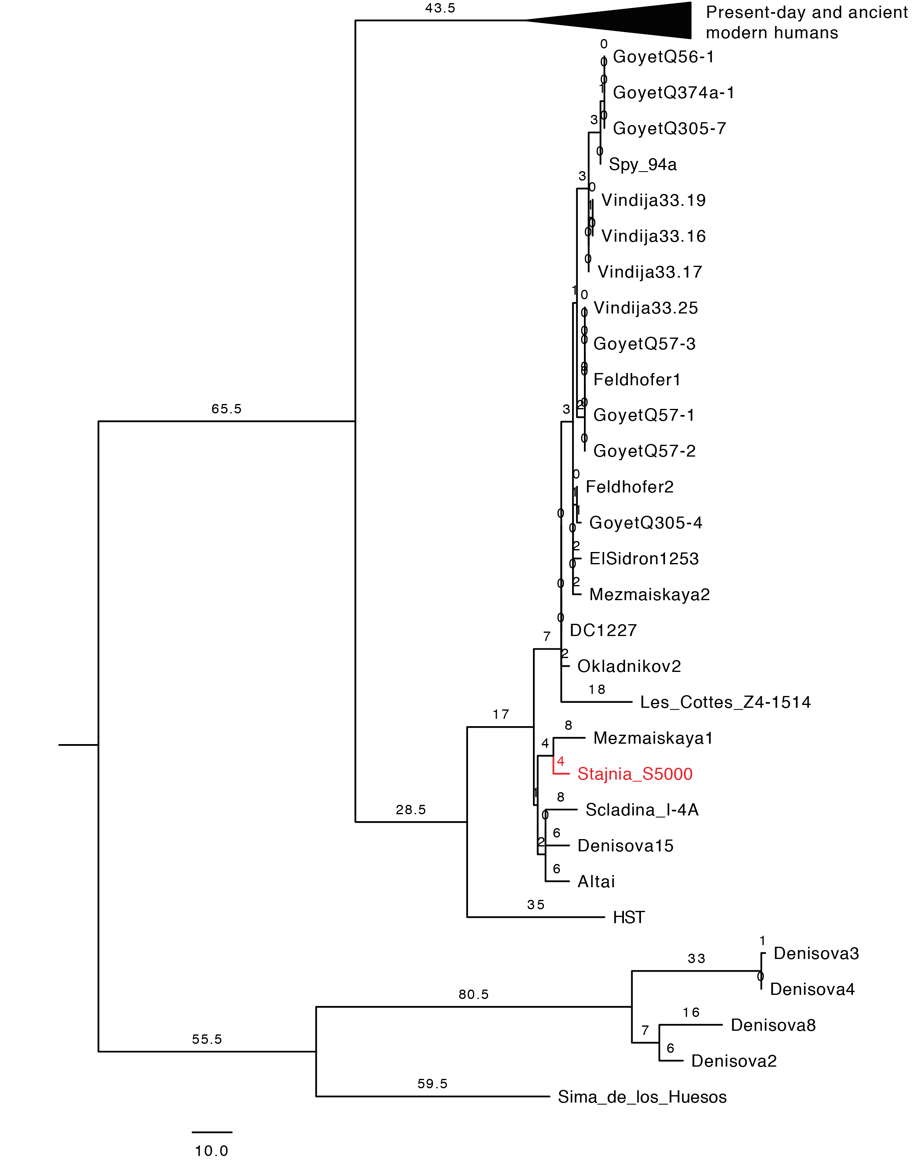
**

**Figure S4:** Map showing the location of Neanderthals’ published genome, listed in Figure 3 - S2, and the extension of the ice sheets during the Weichselian in Fennoscandia, Ireland, Britain and the Alps (modified after ^133^; base map from GeoMappApp (www.geomapapp.org)), MIS 5d – yellow, MIS 5b – orange, MIS 4 – red, early MIS 3 – blue. The glacier extension in other mountain areas is not shown.


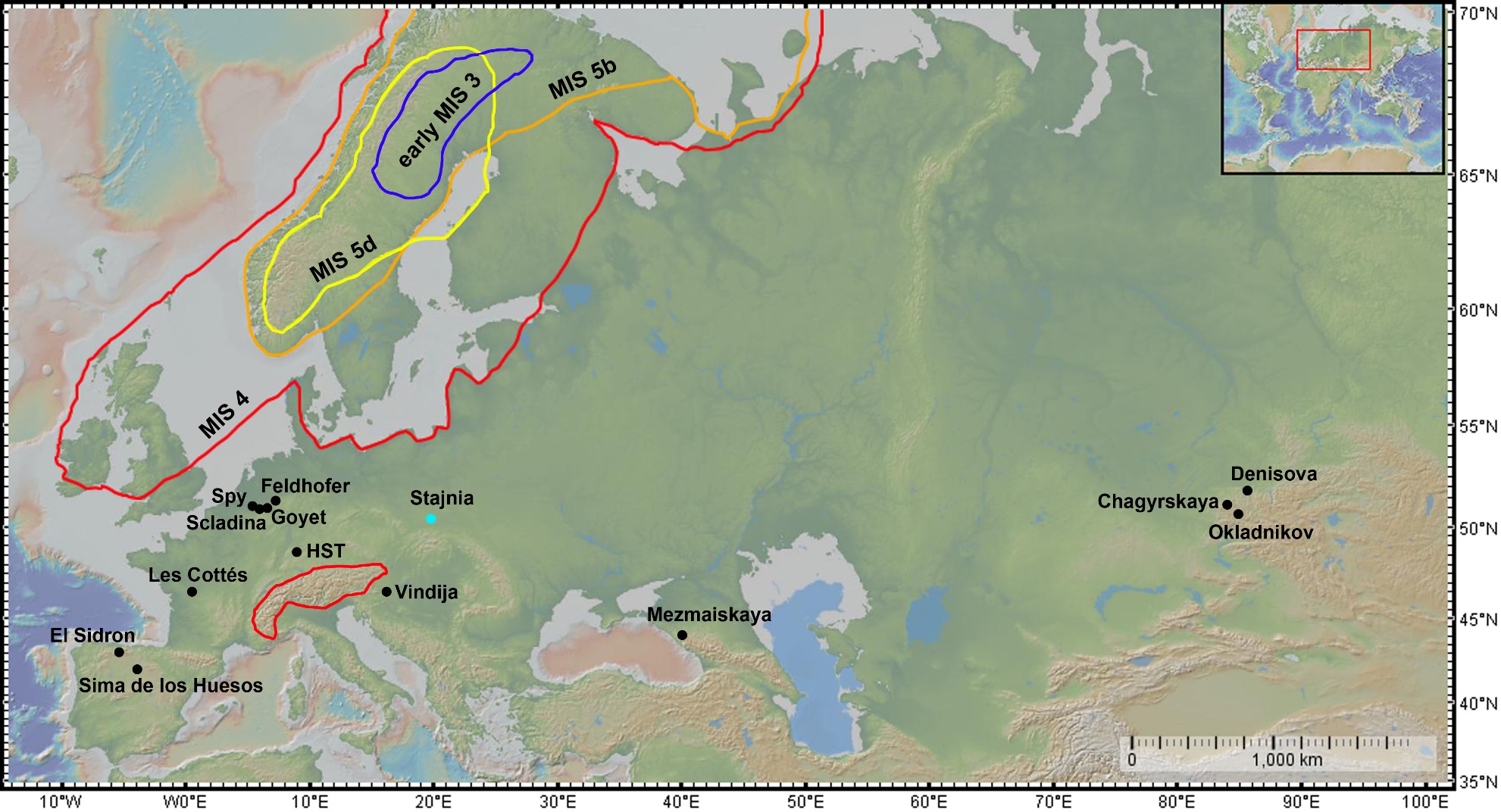


**Table S1**: Radiocarbon dates from previous studies and obtained for this paper are included in the table. For the samples measured at the MPI the elemental analyses were made using a Thermo Scientific Flash Elemental Analyzer, coupled to a Delta V isotope ratio mass spectrometer. The radiocarbon ages were calibrated using the IntCal20 data set ^134^ and OxCal v4.4 ^135^. The calibrated ranges (68.2% and 95.4%) are shown in the table. Previous OSL and U/Th dates are also added in the table.

|  |  |  |  |  |  |  |  |  |  |  | **Modelled (cal BP)** | | | |  |
| --- | --- | --- | --- | --- | --- | --- | --- | --- | --- | --- | --- | --- | --- | --- | --- |
|  |  |  |  |  |  |  |  |  |  |  | ***68.2%*** | | ***95.4%*** | |  |
| **Method** | **MPI**  **Lab Code** | **Level** | **Subm. N°** | **Species** | **Element** | **Collagen %** | **C:N** | **Lab Code** | **Age** | **Err 1s** | *from* | *to* | *from* | *to* | **References** |
| ^14^C | Previous Date | B | unknown | Saiga tatarica | maxilla | unknown | unknown | Poz-28891 | 13500 | 50 | 16360 | 16180 | 16470 | 16080 | ^63^ |
| OSL | Previous Date | B | unknown |  |  |  |  | GdTL-1126 | 8950 | Not reported |  |  |  |  | ^61^ |
| ^14^C | Previous Date | C18 | unknown | Bear | 3d phalanx | unknown | 3.6 | Poz-61719 | 20930 | 140 | 25550 | 25050 | 25680 | 24910 | ^113^ |
| ^14^C | Previous Date | C18 | unknown | Bear | 3d phalanx | unknown | 3.5 | GdA-3894 | 21900 | 90 | 26280 | 26000 | 26370 | 25930 | ^113^ |
| ^14^C | Previous Date | D1 | unknown | Bear | tooth | unknown | unknown | Poz-28892 | >49000 |  |  |  |  |  | ^61^ |
| OSL | Previous Date | D1 | unknown |  |  |  |  | GdTL-1127 | 45900 | Not reported |  |  |  |  | ^61^ |
| ^14^C | Previous Date | D1 | S-23101b | Mammoth | Tusk | 6.9 | 3.1 | OxA-24944 | 44600 | 2100 | 49850 | 45060 | 54890 | 44420 | ^61^ |
| ^14^C | S-EVA 27827 | D1 | S-12182 | UNG3 | LB | 13.2 | 3.2 | MAMS-19879 | 44590 | 690 | 47610 | 46130 | 48470 | 45630 | This paper |
| ^14^C | R-EVA 3213 | D2 | S-5000 | Human | tooth | 5.4 | 3.6 | MAMS-40506 | 22480 | 70 | 27020 | 26490 | 27070 | 26440 | This paper |
| ^14^C | S-EVA 27823 | D2 | S-12305 | UNG5 | LB | 11.7 | 3.2 | MAMS-19878 | >49000 |  |  |  |  |  | This paper |
| U/Th | Previous Date | D2b | unknown | Mammoth | tooth |  |  | W1400 | 52000 | +1900  -1700 |  |  |  |  | ^61^ |
| U/Th | Previous Date | D2b | unknown | Mammoth | tooth |  |  | W1417 | 52000 | +500  -200 |  |  |  |  | ^61^ |
| ^14^C | S-EVA 27814 | D3 | S-12722 | BB | FE | 11.1 | 3.1 | MAMS-19871 | >49000 |  |  |  |  |  | This paper |
| ^14^C | S-EVA 27812 | D3 | S-11572 | UNG3 | LB | 16.6 | 3.2 | MAMS-19869 | >49000 |  |  |  |  |  | This paper |
| ^14^C | S-EVA 27777 | E1 | S-24262 | UNG4 | TI | 10.1 | 3.1 | MAMS-19856 | >49000 |  |  |  |  |  | This paper |

**Table S2**. The metric traits (BL, MD, CBA, and CSI) of the S5000 M^2^ compared to those of the different hominin M^2^ samples.

| **Sample of M^2^s** | **N** | **BL (mm)**  **Mean (SD)** | **Z** | **N** | **MD (mm)**  **Mean (SD)** | **Z** |
| --- | --- | --- | --- | --- | --- | --- |
| **Stajnia S5000 (RM^2^) (a)** | 1 | **11.00** | - | 1 | **10.72*** | - |
| Stajnia S4619 as (RM^2^) (b) | 1 | 12.10 | - | 1 | 11.20 | - |
| Denisovans (c):  DV 4 (as RM^2^ )  DV 8 (as LM^2^) | 2 | 14.68 (-):  14.70  14.65 | - | 2 | 13.70 (-):  13.10  14.30 | - |
| Atapuerca  Sima de los Huesos (d) | 28  (L & R) | 12.18  (0.73) | -2.47 | 28  (L & R) | 9.94  (0.94) | 0.83 |
| Early  Neanderthals (e) | 26  (L & R) | 12.44  (0.83) | -1.74 | 26  (L & R) | 10.98  (1.10) | -0.24 |
| Late Neanderthals (e) | 30  (L & R) | 12.47  (0.98) | -1.50 | 30  (L & R) | 10.52  (0.83) | 0.24 |
| Qafzeh/Skhul  (fossil *H. sapiens*) (f) | 10  (L & R) | 12.00  (0.7) | -1.43 | 10 | 10.5  (1.10) | 0.20 |
| Early Upper Palaeolithic  *H. sapiens* (e) | 12  (L & R) | 12.24  (0.50) | -2.47 | 13  (L & R) | 10.45  (1.07) | 0.25 |
| Late Upper Palaeolithic  *H. sapiens* (e) | 56  (L & R) | 12.18  (0.87) | -1.36 | 56  (L & R) | 10.15  (0.84) | 0.68 |
| Recent *H. sapiens* (e) | 100  (L & R) | 11.15  (0.67) | -0.22 | 100  (L & R) | 9.20  (0.68) | 2.24 |
| **Sample of M^2^s** | **N** | **CBA (mm^2^)**  **Mean (SD)** | **Z** | **N** | **CSI**  **(scale-free)**  **Mean (SD)** | **Z** |
| **Stajnia S5000 (RM^2^)** | 1 | **117.92** | - | 1 | **102.61** | - |
| Denisovans (c):  DV 4 (as RM^2^ )  DV 8 (as LM^2^) | 2 | 201.04 (-):  192.57  209.50 | - | 2 | 107.33 (-):  112.21  102.45 | - |
| Atapuerca  Sima de los Huesos (g) | 6  (L & R) | 136.20  (17.65) | -1.04 | 6  (L & R) | 121.94  (4.12) | -4.70 |
| Neanderthals (h) | 34  (L & R) | 130.37  (17.26) | -0.72 | 34  (L & R) | 118.36  (8.78) | -1.79 |
| Early *H. sapiens* (i) | 7  (L & R) | 133.72  (16.42) | -0.96 | 7  (L & R) | 120.17  (11.42) | -1.54 |
| Upper Palaeolithic  *H. sapiens* (j) | 33  (L & R) | 128.93  (18.88) | -0.58 | 33  (L & R) | 120.14  (6.58) | -2.66 |

**Abbreviations:** * the corrected value of MD diameter; SD – standard deviation; Z – Z score value calculated for metric traits of S5000 tooth using appropriate statistics by subtracting the hominin sample mean value of the metric trait and dividing by the hominin sample standard deviation for this trait (Z score value was calculated when sample N > 2); L – left; R – right; BL – buccoligual crown diameter; MD - mesiodistal crown diameter; the data about the metric traits used in this study were obtained from literature: (a) – ^63^; (b) – ^136^; (c) – ^99^; (d) – ^137^; (e) – ^69^; (f) – ^138^; (g) – ^139,140^ (Atapuerca - Sima de los Huesos specimens); (h) – ^136^ (Stajnia 4619), ^141^ (La Quina 5, Le Moustier, Spy 1, 2, Saccopastore 2, Amud 1), ^142^ (Palomas 1), ^143^ (Shanidar 1, 2, 6), ^144^ (Tabūn), ^145^ (Krapina – 10 individuals); (i) – ^138^ (Misliya -1), ^146^ (Herto), ^144^ (Skhul 5), ^147^ (Qafzeh 6); (j) – ^148^ (Předmostí: I, III, IV, V, VII, IX, X, XIV); ^149^ (Brno III); ^150^ (Dolní Věstonice: 3, 13, 14, 15), ^151,152^ (Mladeč: 1, 2, 8, 47); ^153^ (Sunghir 2, 3). The different techniques of taking the measurements of molar crown: MD and BL may influence of the reliability of the comparisons, but the comparison of these metric traits obtained from different data sources is commonly practiced by scientists (e.g. ^154^).

**Table S3**: Characteristics of the DNA libraries generated from Stajnia S5000 and frequencies of C to T substitutions at the terminal positions of sequence alignments.

| Library ID | Description | Powder used for DNA extraction (mg) | Nr. of molecules in library | Nr. of sequences generated | Nr. of unique mtDNA fragm. | Avr. sequence coverage of the mtDNA genome | Nr. of deaminated mtDNA fragments | Avr. coverage of the mtDNA genome by deaminated fragm. | All fragm. | |
| --- | --- | --- | --- | --- | --- | --- | --- | --- | --- | --- |
|  |  |  |  |  |  |  |  |  | 5’ C→T (%) [observations] | 3’ C → T (%) [observations] |
| A9059 | Stajnia S5000 regular extraction | 40.6 | 4.09E+10 | 754,756 | 13,212 | 76.45 | 2,789 | 7.55 | 15.2  [746/4,908] | 32.9  [1,637/4,977] |
| R5019 | Phosphate washes of tooth powder | 19.8 | 2.55E+09 | 15,940,974 | 53,776 | 180.43 | 14,659 | 45.98 | 46.8  [6,165/13,173] | 36.9  [4,211/11,411] |
| R5020 | Stajnia S5000 extraction after powder pre-treatment |  | 4.31E+08 | 15,459,829 | 5,538 | 17.57 | 1,541 | 4.79 | 42.1  [617/1,465] | 37.5  [423/1,127] |
| R5043 | Phosphate washes of tooth powder |  | 3.65E+09 | 17,156,937 | 60,482 | 207.27 | 17,381 | 55.51 | 45.1  [7,129/15,807] | 39.7  [5,126/12,912] |
| R5044 | Stajnia S5000 extraction after powder pre-treatment |  | 4.35E+08 | 19,996,314 | 4,964 | 15.59 | 1,477 | 4.57 | 44.5  [570/1,281] | 42.3  [445/1,053] |
| A22366 | Stajnia S5000 regular extraction | 10.7 | 1.60E+09 | 313,108 | 78,191 | 228.51 | 26,234 | 77.37 | 48.5 [11,222/23,139] | 38.4 [7,852/20,448] |
| A22367 | Stajnia S5000 regular extraction | 11.0 | 9.88E+08 | 336,790 | 68,814 | 195.95 | 23,324 | 67.21 | 51.2 [9,966/19,465] | 40.3 [7,159/17,764] |
| A22368 | Stajnia S5000 regular extraction | 11.1 | 2.26E+09 | 317,196 | 50,194 | 170.71 | 8,013 | 22.87 | 30.3 [3,390/11,188] | 20.0 [2,502/12,512] |
| A22369 | Stajnia S5000 regular extraction | 11.2 | 1.09E+09 | 306,442 | 58,601 | 181.43 | 13,152 | 37.66 | 35.0 [5,479/15,654] | 27.2 [4,131/15,186] |
| A22370 | Stajnia S5000 regular extraction | 8.8 | 6.88E+08 | 311,996 | 43,737 | 125.46 | 13,624 | 38.86 | 47.9 [5,695/11,890] | 37.0 [4,254/11,498] |
| A9067 | Extraction negative control | - | 1.59E+08 | 103,122 | 354 | 1.26 | 3 | 0.01 | 1.4  [1/69] | 0  [0/86] |
| R5050 | Extraction negative control | - | 2.66E+07 | 81,198 | 154 | 0.46 | 4 | 0.01 | 0  [0/35] | 3.2  [1/31] |
| A9070 | Library negative control | - | 1.71E+08 | 98,447 | 30 | 0.1 | - | - | 0  [0/7] | 0  [0/5] |
| R5051 | Library negative control | - | 1.36E+07 | 51,856 | 19 | 0.05 | - | - | 0  [0/2] | 0  [0/5] |

Nr – number; Avr – Average; fragm. – fragments

**Table S4:** Phylogenetic assignment of mitochondrial DNA fragments overlapping 63 diagnostic positions, at which 311 present-day human mtDNA sequences differ from those of 18 Neanderthals. Results are shown for all fragments and only those fragments with terminal C to T substitutions (deaminated fragments).

| **Library ID** | **Description** | **All fragments** | | **Deaminated fragments** | |
| --- | --- | --- | --- | --- | --- |
|  |  | **%Neanderthal [observations]** | **%Human [observations]** | **%Neanderthal [observations]** | **%human [observations]** |
| A9059 | Stajnia S5000 regular extraction | 57.98  [923/1,592] | 42.02  [669/1,592] | 100  [98/98] | 0  [0/98] |
| R5019 | Phosphate washes of tooth powder | 66.64 [2,407/3,612] | 33.36 [1,205/3,612] | 88.75  [568/640] | 11.25  [72/640] |
| R5020 | Stajnia S5000 extraction after powder pre-treatment | 69.01  [1,335/1,441] | 30.9  [106/1,441] | 90.11  [74/91] | 9.89  [9/91] |
| R5043 | Phosphate washes of tooth powder | 61.89  [2,537/4,099] | 38.11 [1,562/4,099] | 84.73  [649/766] | 15.27  [117/766] |
| R5044 | Stajnia S5000 extraction after powder pre-treatment | 70.34  [204/290] | 29.66  [86/290] | 85.54  [59/83] | 14.46  [12/83] |
| A22366 | Stajnia S5000 regular extraction | 90.82 [3,095/3,408] | 9.18 [313/3,408] | 99.15 [931/939] | 0.85 [8/939] |
| A22367 | Stajnia S5000 regular extraction | 97.02 [2,934/3,024] | 2.98 [90/3,024] | 99.27 [815/821] | 0.73 [6/821] |
| A22368 | Stajnia S5000 regular extraction | 24.37 [975/4,001] | 75.63 [3,026/4,001] | 92.22 [249/270] | 7.78 [21/270] |
| A22369 | Stajnia S5000 regular extraction | 44.65 [1,604/3,592] | 55.35 [1,988/3,592] | 94.68 [463/489] | 5.32 [26/489] |
| A22370 | Stajnia S5000 regular extraction | 84.24 [1,758/2,087] | 15.76 [329/2,087] | 98.66 [517/524] | 1.34 [7/524] |
| A9067 | Extraction negative control | 0  [0/32] | 100  [32/32] | 0  [0/0] | 0  [0/0] |
| R5050 | Extraction negative control | 0  [0/21] | 100  [21/21] | 0  [0/0] | 0  [0/0] |
| A9070 | Library negative control | 0  [0/5] | 100  [5/5] | 0  [0/0] | 100  [1/1] |
| R5051 | Library negative control | 0  [0/0] | 0  [0/0] | 0  [0/0] | 0  [0/0] |

**Table S5:** Unresolved positions in the mitochondrial consensus sequence of Stajnia S5000.

| Position in rCRS coordinates | Coverage | Consensus support |
| --- | --- | --- |
| 191 | 2 |  |
| 193 | 2 |  |
| 194 | 1 |  |
| 195 | 1 |  |
| 196 | 1 |  |
| 310 | 130 | 58.5% |
| 8,468 | 13 | 61.5% |
| 16,129 | 58 | 51.7% |
| 16,148 | 25 | 60.0% |
| 16,171 | 12 | 50.0% |
| 16,209 | 17 | 52.9% |
| 16,278 | 17 | 52.9% |
| 16,304 | 9 | 66.7% |
| 16,320 | 17 | 52.9% |

**Table S6:** Frequencies of C to T substitutions at the terminal positions of DNA sequences identified as originating from present-day humans or Neanderthals.

| **Description** | **Human sequences** | | **Neanderthal sequences** | |
| --- | --- | --- | --- | --- |
|  | **5’ C→T (%) [observations]** | **3’ C → T (%) [observations]** | **5’ C→T (%) [observations]** | **3’ C → T (%) [observations]** |
| Stajnia S5000  all libraries merged | **7.0** [304/4,331] | **4.6** [205/4,462] | **48.2**  [4,086/8,477] | **38.4** [2,796/7,281] |

**Table S7**: Mitochondrial genomes of ancient modern humans and Neanderthals and their respective radiocarbon dates that were used for Bayesian phylogenetic analyses. All radiocarbon dates were calibrated using IntCal13 ^155^ and OxCal v4.3 ^156^.

| **Individual** | **mtDNA accession number** | **Date** | **95.4% confidence intervals** | **Reference** |
| --- | --- | --- | --- | --- |
| ***Ancient modern humans*** | | | | |
| Ust‘-Ishim | - | 45,045 | 43,212-46,878 | ^83^ |
| Tianyuan | KC417443 | 39,008 | 37,761-40,254 | ^85^ |
| Kostenki 14 | FN600416 | 37,473 | 36,262-38,684 | ^75^ |
| Dolní Věstonice 13 | KC521459 | 31,071 | 30,884-31,249 | ^82,157^ |
| Dolní Věstonice 14 | KC521458 | 30,934 | 30,741-31,120 | ^82,157^ |
| Oberkassel 998 | KC521457 | 14,077 | 13,755-14,105 | ^82^ |
| Boshan 11 | KC521454 | 8,234 | 8,152-8,316 | ^82^ |
| Loschbour | KC521455 | 8,054 | 7,948-8,160 | ^82^ |
| Iceman | EU810403 | 5,300 | 5,275,-5,325 | ^84^ |
| Saqqaq Eskimo | EU725621 | 4,504 | 4,423-4,585 | ^86^ |
| ***Neanderthals*** | | | | |
| Goyet Q305-4 | KX198087 | 44,236 | 43,386-45,085 | ^94^ |
| Mezmaiskaya 2 | MG025537 | 43,834 | 42,038-45,630 | ^87,158^ |
| Feldhofer 1 | FM865407 | 43,707 | 42,670-44,744 | ^90,159^ |
| Vindija 33.16 | AM948965 | 43,707 | 39,234-48,179 | ^80,160^ |
| Feldhofer 2 | FM865408 | 43,268 | 42,193-44,342 | ^90,159^ |
| Les Cottés Z4-1514 | MG025536 | 43,230 | 42,720-43,740 | ^87^ |
| Goyet Q56-1 | KX198082 | 42,515 | 42,03-42,967 | ^94^ |
| Goyet Q57-3 | KX198083 | 42,407 | 41,964-42,867 | ^94^ |
| Goyet Q57-2 | KX198088 | 41,185 | 40,595-41,775 | ^94^ |
| Spy 94a | MG025538 | 40,463 | 39,234-48,179 | ^87,161^ |

**Table S8**: Estimated molecular ages of Neanderthal specimens and divergence times. The estimates are based on the coding region only and from three independent MCMC runs with 75,000,000 iterations.

| **Individual** | **Mean value** | **95% HPD lower** | **95% HPD upper** | **ESS** |
| --- | --- | --- | --- | --- |
| Hohlenstein-Stadel | 122,950 | 69,236 | 186,830 | 3,097 |
| Altai Neandertal | 132,430 | 94,378 | 170,950 | 1,058 |
| Denisova 15 | 120,680 | 80,812 | 168,150 | 1,084 |
| Scladina I-4A | 121,520 | 81,699 | 160,870 | 1,173 |
| ***Stajnia S5000*** | ***116,609*** | ***83,101*** | ***152,515*** | ***1,349*** |
| Mezmaiskaya 1 | 98,831 | 62,159 | 135,496 | 1,567 |
| Okladnikov 2 | 92,891 | 68,293 | 117,657 | 1,910 |
| Denisova 11 (DC1227) | 99,867 | 76,681 | 122,792 | 1,451 |
| El Sidron 1253 | 57,515 | 42,731 | 74,704 | 6,904 |
| Vindija 33.17 | 49,681 | 42,538 | 57,801 | 8,316 |
| Vindija 33.19 | 44,969 | 37,442 | 51,950 | 8,289 |
| Vindija 33.25 | 43,912 | 35,397 | 52,991 | 7,982 |
| Goyet Q305-7 | 40,677 | 33,564 | 45,643 | 19,461 |
| Goyet Q374a-1 | 40,663 | 33,640 | 45,712 | 19,086 |
| **TMRCAs** | **Mean value** | **95% HPD lower** | **95% HPD upper** |  |
| Neanderthal – modern human TMRCA | 419,091 | 367,751 | 468,857 |  |
| Neanderthal TMRCA | 276,000 | 233,369 | 320,263 |  |
| Altai Neanderthal (Denisova 5), Scladina I-4A, Denisova 15, Mezmaiskaya 1 and Stajnia S5000 TMRCA | 170,030 | 138,954 | 202,865 |  |
| Mezmaiskaya 1, Stajnia S5000, Okladnikov 2, Denisova 11 and later Neanderthals TMRCA | 152,168 | 123,800 | 181,918 |  |

**Table S9**: Number of Identified Specimens (NISP) and Minimal Numbers of Individuals (MNI) of the carnivores and ungulates of Stajnia Cave divided by stratigraphic units.

| **Taxon** | **Unit E1**  **(MIS 4)**  **NISP/MNI** | **Unit E1/D**  **(MIS 4/3)**  **NISP/MNI** | **Unit D**  **(MIS 3)**  **NISP/MNI** | **Unit D/C**  **(MIS 3/2)**  **NISP/MNI** | **Unit C**  **(MIS 2)**  **NISP/MNI** | **Unit C/A**  **(MIS 2/1)**  **NISP/MNI** | **Unit A**  **(MIS 1)**  **NISP/MNI** | **Dump**  **NISP/MNI** | **Unknown**  **NISP/MNI** |
| --- | --- | --- | --- | --- | --- | --- | --- | --- | --- |
| *Canis lupus* sp. | 6/2 | 2/1 | 34/3 | 13/5 | 11/2 | 2/1 | 1/1 | 4/1 | 2/1 |
| *Vulpes vulpes* ^162^ | 14/2 | 11/2 | 56/4 | 219/11 | 141/8 | 15/2 | 7/1 | 16/3 | 13/3 |
| *Vulpes lagopus* ^162^ | 3/1 | 2/1 | 47/8 | 198/12 | 166/10 | 2/1 |  | 5/2 | 3/1 |
| *Ursus spelaeus ingressus* ^163^ | 27/4 | 4/2 | 143/7 | 44/5 | 3/1 |  |  | 6/1 | 11/4 |
| *Ursus arctos priscus* s. l. | 1/1 | 2/1 | 1/1 | 3/2 | 2/2 |  |  |  |  |
| *Ursus arctos arctos* ^162^ |  |  |  |  |  | 2/1 | 2/1 | 1/1 | 2/1 |
| *Ursus* sp. | 187 | 15 | 314 | 947 | 16 | 5 | 8 | 5 | 11 |
| *Gulo gulo* ^162^ | 1/1 | 1/1 | 1/1 | 2/1 | 1/1 |  |  |  |  |
| *Meles meles* ^162^ | 1/1 | 2/1 | 1/1 | 2/2 | 1/1 | 1/1 | 2/1 | 1/1 |  |
| *Martes martes* ^162^ | 22/3 | 5/2 | 3/1 | 5/2 | 2/1 | 4/1 | 2/1 | 2/1 |  |
| *Mustela eversmanii* ^164^ |  |  | 1/1 | 1/1 | 1/1 |  |  |  |  |
| *Mustela putorius* ^162^ | 1/1 | 1/1 | 3/1 | 2/2 | 25/2 | 14/3 | 2/1 | 1/1 | 3/1 |
| *Mustela erminea* ^162^ | 19/6 | 85/25 | 196/18 | 112/13 | 59/6 | 12/3 | 18/4 | 5/2 | 4/2 |
| *Mustela nivalis* ^165^ | 25/10 | 7/3 | 345/66 | 297/138 | 64/25 | 14/3 | 27/9 | 11/3 | 12/5 |
| *Panthera spelaea spelaea* ^166^ |  |  | 2/1 |  |  |  |  |  | 1/1 |
| *Lynx lynx* ^162^ | 1/1 |  | 1/1 |  | 1/1 | 1/1 |  |  |  |
| *Felis silvestris* ^167^ | 2/1 | 1/1 | 12/2 | 4/2 | 3/1 | 1/1 | 5/2 | 2/1 | 3/1 |
| *Crocuta crocuta spelaea* ^168^ | 4/2 | 3/2 | 18/3 | 9/2 |  |  |  | 2/1 | 3/1 |
| *Mammuthus primigenius* ^169^ | - | - | - | 1/1 | 2/1 | 1/1 | - | 4/1 | - |
| *Coelodonta antiquitatis* ^170^ | - | - | 5/1 | 1/1 | 3/1 | 1/1 | - | - | 2/2 |
| *Equus ferus* ^171^ | - | - | 1/1 | 4/1 | 4/1 | 5/1 | - | 5/2 | - |
| *Sus scrofa* ^162^ | - | - | - | - | - | - | - | 7/2 | - |
| *Cervus elaphus* ^162^ | - | - | - | 1/1 | 1/1 | - | - | 3/1 | - |
| *Rangifer tarandus* ^162^ | 7/1 | 2/1 | 126/7 | 4/1 | 98/4 | 26/2 | 2/1 | 64/5 | 6/4 |
| *Bison priscus* ^172^ | - | - | - | 4/1 | 13/3 | 38/3 | - | 9/1 | - |
| *Saiga tatarica* ^165^ | - | - | - | - | - | 2/1 | - | 1/1 | - |
| *Ovis/Capra* | - | - | - | - | - | - | - | 2/1 | - |

**Table S10**: Preliminary number and percentage of the lithic assemblages (>2cm) of the Middle Palaeolithic layers of Stajnia Cave.

|  | **D1** | | **D2** | | **D3** | | **E1** | | **E2** | | **Total** | |
| --- | --- | --- | --- | --- | --- | --- | --- | --- | --- | --- | --- | --- |
|  | **N** | ***%*** | **N** | ***%*** | **N** | ***%*** | **N** | ***%*** | **N** | ***%*** | **N** | ***%*** |
| Cortical flake | 9 | *4.0* | 3 | *3.9* |  |  |  |  |  |  | 12 | *3.6* |
| Ordinary flake | 25 | *11.2* | 10 | *13.0* | 2 | *20* |  |  | 1 | *20* | 38 | *11.5* |
| Predetermining Lev. flake | 2 | *0.9* |  |  |  |  |  |  |  |  | 2 | *0.6* |
| Levallois rec. uni. flake | 3 | *1.3* |  |  |  |  |  |  |  |  | 3 | *0.9* |
| Levallois rec. bid. flake | 2 | *0.9* |  |  |  |  |  |  |  |  | 2 | *0.6* |
| Levallois rec. centr. flake |  | *0.0* | 1 | *1.3* |  |  |  |  |  |  | 1 | *0.3* |
| Levallois point | 1 | *0.4* |  |  |  |  |  |  |  |  | 1 | *0.3* |
| Levallois flake undeter. | 2 | *0.9* | 2 | *2.6* |  |  |  |  |  |  | 4 | *1.2* |
| Core-edge removal flake | 5 | *2.2* | 3 | *3.9* |  |  |  |  | 1 | *20* | 9 | *2.7* |
| Pseudo-Levallois point | 1 | *0.4* |  |  |  |  |  |  |  |  | 1 | *0.3* |
| Unidirectional flake | 11 | *4.9* | 4 | *5.2* | 1 | *10* |  |  |  |  | 16 | *4.8* |
| Centripetal flake | 13 | *5.8* | 3 | *3.9* |  |  |  |  |  |  | 16 | *4.8* |
| Orthogonal flake | 1 | *0.4* |  |  |  |  |  |  |  |  | 1 | *0.3* |
| Reshaping flaking surface | 1 | *0.4* | 2 | *2.6* |  |  |  |  |  |  | 3 | *0.9* |
| Kombewa-type flake | 2 | *0.9* |  |  |  |  |  |  |  |  | 2 | *0.6* |
| Bifacial shaping flake | 1 | *0.4* | 1 | *1.3* |  |  |  |  |  |  | 2 | *0.6* |
| Knapping accident | 1 | *0.4* | 1 | *1.3* |  |  |  |  |  |  | 2 | *0.6* |
| Flake frag. | 59 | *26.5* | 21 | *27.3* | 5 | *50* | 8 | *50* | 2 | *40* | 95 | *28.8* |
| Debris | 7 | *3.1* | 4 | *5.2* |  |  |  |  |  |  | 11 | *3.3* |
| Preform of bifacial tool | 5 | *2.2* |  |  |  |  | 1 | *6.25* |  |  | 6 | *1.8* |
| Bifacial tool | 3 | *1.3* | 1 | *1.3* |  |  |  |  |  |  | 4 | *1.2* |
| Bifacial tool frag. | 2 | *0.9* | 1 | *1.3* |  |  |  |  |  |  | 3 | *0.9* |
| Leaf point frag. | 1 | *0.4* |  |  |  |  |  |  |  |  | 1 | *0.3* |
| Scrapers | 12 | *5.4* | 3 | *3.9* |  |  |  |  | 1 | *20* | 16 | *4.8* |
| Groszak | 1 | *0.4* |  |  |  |  |  |  |  |  | 1 | *0.3* |
| Notched tool | 3 | *1.3* |  |  |  |  |  |  |  |  | 3 | *0.9* |
| Point | 1 | *0.4* |  |  |  |  |  |  |  |  | 1 | *0.3* |
| Retouched tool frag. | 9 | *4.0* | 2 | *2.6* |  |  | 1 | *6.25* |  |  | 12 | *3.6* |
| Core | 38 | *17.0* | 15 | *19.5* | 2 | *20* | 5 | *31.25* |  |  | 60 | *18.2* |
| Tested pebbles | 2 | *0.9* |  |  |  |  |  |  |  |  | 2 | *0.6* |
| **Total** | 223 | *100* | 77 | *100* | 10 | *100* | 15 | *100* | 5 | *100* | 330 | *100* |

**Table S11**: Preliminary number and percentage of the core assemblages of the Middle Palaeolithic layers of Stajnia Cave.

|  | **D1** | | **D2** | | **D3** | | **E1** | | **Total** | |
| --- | --- | --- | --- | --- | --- | --- | --- | --- | --- | --- |
|  | **N** | ***%*** | **N** | ***%*** | **N** | ***%*** | **N** | ***%*** | **N** | ***%*** |
| Levallois | 1 | *2.6* | 1 | *6.7* | 1 | *50* |  |  | 3 | *5* |
| Discoid | 8 | *21.1* | 4 | *26.7* | 1 | *50* | 2 | *40* | 15 | *25.0* |
| Hierarchized uni. | 1 | *2.6* | 1 | *6.7* |  |  |  |  | 2 | *3.3* |
| Hierarchized bid. | 4 | *10.5* |  |  |  |  |  |  | 4 | *6.7* |
| Hierarchized centr. | 6 | *15.8* | 1 | *6.7* |  |  |  |  | 7 | *11.7* |
| Unidirectional | 8 | *21.1* | 4 | *26.7* |  |  | 2 | *40* | 14 | *23.3* |
| Orthogonal |  |  | 1 | *6.7* |  |  |  |  | 1 | *1.7* |
| Centripetal |  |  | 1 | *6.7* |  |  | 1 | *20* | 2 | *3.3* |
| Polyhedral | 1 | *2.6* | 1 | *6.7* |  |  |  |  | 2 | *3.3* |
| Core-on-flake | 4 | *10.5* | 1 | *6.7* |  |  |  |  | 5 | *8.3* |
| Fragment | 5 | *13.2* |  |  |  |  |  |  | 5 | *8.3* |
| **Total** | 38 | *100* | 15 | *100* | 2 | *100* | 5 | *100* | 60 | *100* |

**SI References**

1 Fletcher, W. J. *et al.* Millennial-scale variability during the last glacial in vegetation records from Europe. *Quaternary Sci. Rev.* **29**, 2839-2864, doi:10.1016/j.quascirev.2009.11.015 (2010).

2 Wohlfarth, B. A review of Early Weichselian climate (MIS 5d-a) in Europe. *Technical report/Svensk kärnbränslehantering AB* **44** (2013).

3 Kahlke, R.-D. The origin of Eurasian Mammoth Faunas (Mammuthus–Coelodonta Faunal Complex). *Quaternary Sci. Rev.* **96**, 32-49, doi:https://doi.org/10.1016/j.quascirev.2013.01.012 (2014).

4 Gaudzinski-Windheuser, S. & Roebroeks, W. in *Neanderthal Lifeways, Subsistence and Technology* *Vertebrate Paleobiology and Paleoanthropology Series* (eds N. J. Conard & J. Richter) 61-71 (Springer, 2011).

5 Gamble, C. in *The Pleistocene Old World: Regional Perspectives* (ed Olga Soffer) 81-98 (Springer US, 1987).

6 Hosfield, R. Walking in a Winter Wonderland? Strategies for Early and Middle Pleistocene Survival in Midlatitude Europe. *Curr. Anthropol.* **57**, 653-682, doi:10.1086/688579 (2016).

7 Jöris, O. in *Axe Age. Acheulian Tool-making from Quarry to Discard* (eds N. Goren-Imbar & G. Sharon) 287-310 (Equinox Publishing, 2006).

8 Yevtushenko, A. The industries of the Eastern Micoquian: some approaches to typological variability. *Préhistoire d’Anatolie, Genèse de deux mondes I. ERAUL* **85**, 113e123 (1998).

9 Bosinski, G. in *Fundamenta A/4* (Böhlau-Verlag, 1967).

10 Mania, D. *Auf den Spuren des Urmenschen. Die Funde von Bilzingsleben*. (Deutscher Verlag der Wissenschaften GmbH, 1990).

11 Veil, S. *et al.* Ein mittelpaläolithischer Fundplatz aus der Weichsel-Kaltzeit bei Lichtenberg, Lkr. Lüchow-Dannenberg. *Germania* **72**, 1-66 (1994).

12 Blaser, F., Bourguignon, L., Sellami, F. & Rios Garaizar, J. Une série lithique à composante Laminaire dans le Paléolithique moyen du Sud-Ouest de la France : le site de Cantalouette 4 (Creysse, Dordogne, France). *Bulletin de la Société préhistorique française* **109**, 5-33 (2012).

13 Farizy, C. & Tuffreau, A. Industries et cultures du Paléolithique moyen récent dans la moitié Nord de la France. *Chronostratigraphie et faciès culturels du Paléolithique inférieur et moyen dans l’Europe du Nord-Ouest*, 225-234 (1986).

14 Frick, J. A., Herkert, K., Hoyer, C. T. & Floss, H. The performance of tranchet blows at the Late Middle Paleolithic site of Grotte de la Verpillière I (Saône-et-Loire, France). *PLOS ONE* **12**, e0188990, doi:10.1371/journal.pone.0188990 (2017).

15 Gábori, M. *Les civilisations du paléolithique moyen entre les Alpes et l'Oural: esquisse historique*. (Akadémiai Kiadó, 1976).

16 Golovanova, L. V., Doronicheva, E. V., Doronichev, V. B. & Shirobokov, I. G. Bifacial scraper-knives in the Micoquian sites in the North-Western Caucasus: Typology, technology, and reduction. *Quatern. Int.* **428**, 49-65, doi:https://doi.org/10.1016/j.quaint.2015.12.069 (2017).

17 Kozłowski, J. K. Middle Palaeolithic variability in Central Europe: Mousterian vs Micoquian. *Quatern. Int.* **326–327**, 344-363, doi:http://dx.doi.org/10.1016/j.quaint.2013.08.020 (2014).

18 Gladilin, V. N. in *Arkheologia UkrSSR* Vol. 1 12-54 (Naukova dumka, 1985).

19 Koulakovskaya, L. Aspects typologiques des industries micoquiennes : le site de Korolevo en Ukraine. *Paléo, Revue d'Archéologie Préhistorique*, 207-211 (1995).

20 Bárta, J. Mittelpaläolithische Funde im Gebiet der Slowakei. *Ethnographisch-archaologische Zeitschrift* **31**, 122-134 (1990).

21 Neruda, P. *Middle Palaeolithic in Moravian Caves*. 249 (Muni Press, 2011).

22 Valoch, K. *Die Erforschung der Kůlna-Höhle 1961-1976*. (Moravské muzeum-Anthropos Institut, 1988).

23 Wiśniewski, A. *et al.* Looking for provisioning places of shaped tools of the late Neanderthals: A study of a Micoquian open-air site, Pietraszyn 49a (southwestern Poland). *Comptes Rendus Palevol* **18**, 367-389, doi:https://doi.org/10.1016/j.crpv.2019.01.003 (2019).

24 Budek, A., Kalicki, T., Kaminská, L. u., Kozłowski, J. K. & Mester, Z. Interpleniglacial profiles on open-air sites in Hungary and Slovakia. *Quatern. Int.* **294**, 82-98, doi:https://doi.org/10.1016/j.quaint.2012.02.022 (2013).

25 Frick, J. A. & Floss, H. Analysis of bifacial elements from Grotte de la Verpillière I and II (Germolles, France). *Quatern. Int.* **428**, 3-25, doi:https://doi.org/10.1016/j.quaint.2015.10.090 (2017).

26 Gouédo, J.-M. *Le technocomplexe micoquien en Europe de l'ouest et centrale: exemples de trois gisements du sud-est du basin parisien, Vinneuf et Champlost (Yonne), Verrières-le-Buisson (Essonne)* PhD thesis thesis, Lille 1, (1999).

27 Marcy, J.-L. in *Paleolithique et Mesolithique du nord de la France, nouvelles recherches 2* (ed A. Tuffreau) 103-111 (Universite des Sciences et Technologies de Lille, 1991).

28 Cyrek, K., Sudoł, M., Czyżewski, Ł., Osipowicz, G. & Grelowska, M. Middle Palaeolithic cultural levels from Middle and Late Pleistocene sediments of Biśnik Cave, Poland. *Quatern. Int.* **326–327**, 20-63, doi:http://dx.doi.org/10.1016/j.quaint.2013.12.014 (2014).

29 Monnier, G. F. The Lower/Middle Paleolithic periodization in Western Europe. *Curr. Anthropol.* **47**, 709-744 (2006).

30 Richter, J. *Der G-Schichten-Komplex der Sesselfelsgotte - Zum Verständnis des Micoquien*. Vol. 7 473 (SDV, 1997).

31 Boëda, E. Steinartefakt-Produktionssequenzen im Micoquien der Kulna-Höhle. *Quartär* **45/46**, 75-98 (1995).

32 Bordes, F. Mousterian Cultures in France. *Science* **134**, 803-810 (1961).

33 Burdukiewicz, J. M. in *Toward Modern Humans. The Yabrudian and Micoquian. Proceedings of a Congress help at the University of Haifa, Novemebr 3-9, 1996* (eds A. Ronen & M. Weinstein-Evron) 155-165 (BAR International Series 850, 2000).

34 Chmielewski, W. Ensembles micoquo-prondnikiens en Europe Centrale. *Geographia Polonica* **17**, 371-386 (1969).

35 Kowalski, S. Ciekawsze zabytki paleolityczne z najnowszych badań archeologicznych (1963-1965) w Jakini Ciemnej w Ojcowie, pow. Olkusz. *Materiały Archeologiczne* **8**, 39-46 (1967).

36 Krukowski, S. in *Prehistoria ziem polskich* (eds S. Krukowski & J. Kostrzewski) 1-117 (Polska Akademia Umiejetnosci, 1939).

37 Jöris, O. Pradniktechnik im Micoquien der Balver Höhle. *Archäologisches Korrespondenzblatt* **22**, 1-12 (1992).

38 Urbanowski, M. *Pradnik knives as an element of Micoquian techno-stylistic specifics*, Warsaw University (2003).

39 Chabai, V., Richter, J. & Uthmeier, T. *Kabazi II: Last Interglacail occupation, environment & subsistence*. (Shlyakh, 2005).

40 Chabai, V. P. in *The Black Sea Flood Question: Changes in Coastline, Climate, and Human Settlement* (eds Valentina Yanko-Hombach, Allan S. Gilbert, Nicolae Panin, & Pavel M. Dolukhanov) 279-296 (Springer Netherlands, 2007).

41 Golovanova, L. V. Les hommes de Néandertal du Caucase du Nord : entre l’Ouest et l’Est. *L'Anthropologie* **119**, 254-301, doi:https://doi.org/10.1016/j.anthro.2015.04.003 (2015).

42 Paunescu, A. *Ripiceni-Izvor. Paleolitic si Mezolitic*. (Editura Academiei Romane, 1993).

43 Ocherednoi, A., Salnaya, N., Voskresenskaya, E. & Vishnyatsky, L. New geoarcheological studies at the Middle Paleolithic sites of Khotylevo I and Betovo (Bryansk oblast, Russia): Some preliminary results. *Quatern. Int.* **326-327**, 250-260, doi:https://doi.org/10.1016/j.quaint.2013.11.005 (2014).

44 Marks, A. E. & Chabai, V. P. in *Transitions Before the Transition* (eds E. Hovers & S. L. Kuhn) 121-135 (Springer, 2006).

45 Chabai, V. P. in *Neanderthals and modern humans-discussing the transition: Central and Eastern Europe from 50,000-30,000 B.P.* Vol. Neanderthal Museum (eds J. Orschiedt & G.-C. Weniger) 196-211 (Wissenschaftliche Schriften des Neanderthal Museums, 2000).

46 Demidenko, Y. Palaeolithic industries with bifacial technologies and Crimean Micoquian Tradition as one of their Middle Palaeolithic industrial examples. *Litikum* **3** (2015).

47 Banesz, L. Mittelpaläolithische kleinformige Industrie aus den Travertinfundstellen der Zips. *Slovenská archeológia* **38**, 45-88 (1990).

48 Richter, J. in *Neanderthals in Europe* Vol. 117 (eds B. Demarsin & M. Otte) 51-66 (ERAUL, 2006).

49 Uthmeier, T. *Micoquien, Aurignacien und Gravettien in Bayern. Eine regionale Studie zum Übergang vom Mittel- zum Jungpaläolithikum.*, (Dr. Rudolf Habelt, 2004).

50 Binford, L. R. in *The explanation of culture change: models in prehistory* (ed C. Renfrew) 227-254 (Duckwoth, 1973).

51 Bordes, F. & de Sonneville-Bordes, D. The Significance of Variability in Palaeolithic Assemblages. *World Archaeology* **2**, 61-73 (1970).

52 Gladilin, V. in *Proceedings of the VIIth International Congress of Proto-and Prehistorians.* 14-18.

53 Ruebens, K. Regional behaviour among late Neanderthal groups in Western Europe: A comparative assessment of late Middle Palaeolithic bifacial tool variability. *J Hum. Evol.* **65**, 341-362, doi:http://dx.doi.org/10.1016/j.jhevol.2013.06.009 (2013).

54 Picin, A. Short-term occupations at the lakeshore: a technological reassessment of the open–air site Königsaue (Germany). *Quartär*, 7-32, doi:10.7485/QU63_1 (2016).

55 Wiśniewski, A. in *Erkenntnis-Jäger. Kultur und Umwelt des frühen Menschen. Festschrift Für Dietrich Mania, Veröffentlichungen des Landesamtes für Archäologie* Vol. 57 (eds J.M. Burdukiewicz *et al.*) 679-688 (Landesmuseum für Vorgeschichte--Sachsen-Anhalt, 2003).

56 Wiśniewski, A. *et al.* Occupation dynamics north of the Carpathians and Sudetes during the Weichselian (MIS5d-3): The Lower Silesia (SW Poland) case study. *Quatern. Int.* **294**, 20-40, doi:http://dx.doi.org/10.1016/j.quaint.2011.09.016 (2013).

57 Chabai, V., Richter, D. & Uthmeier, T. *Kabazi V: Interstratification of Micoquian & Levallois-Mousterian Camp Sites. Palaeolithic sites of Crimea*. (Shlyakh, 2008).

58 Doronicheva, E. V., Kulkova, M. A. & Shackley, S. M. Raw Material Exploitation, Transport, and Mobility in the Northern Caucasus Eastern Micoquian. *PaleoAnthropology*, 1-45 (2016).

59 Uthmeier, T. & Chabai, V. in *Settlement Dynamics of the Middle Paleolithic and Middle Stone Age* Vol. 3 (eds N. Conard & A. Delagnes) 195-234 (Kerns Verlag, 2010).

60 Valde-Nowak, P. & Cieśla, M. in *Short-Term Occupations in Paleolithic Archaeology: Definition and Interpretation* (eds João Cascalheira & Andrea Picin) 105-120 (Springer International Publishing, 2020).

61 Żarski, M. *et al.* Stratigraphy and palaeoenvironment of Stajnia Cave (southern Poland) with regard to habitation of the site by Neanderthals. *Geological Quarterly* **61**, 350-369, doi: 310.7306/gq. 1355 (2017).

62 Nadachowski, A., Lipecki, G., Ratajczak, U., Stefaniak, K. & Wojtal, P. Dispersal events of the saiga antelope (Saiga tatarica) in Central Europe in response to the climatic fluctuations in MIS 2 and the early part of MIS 1. *Quatern. Int.* **420**, 357-362, doi:https://doi.org/10.1016/j.quaint.2015.11.068 (2016).

63 Urbanowski, M. *et al.* The first Neanderthal tooth found north of the Carpathian Mountains. *Die Naturwissenschaften* **97**, 411-415 (2010).

64 Molnar, S. Human tooth wear, tooth function and cultural variability. *American Journal of Physical Anthropology* **34**, 175-189, doi:10.1002/ajpa.1330340204 (1971).

65 Bailey, S. E. *Neandertal dental morphology: implications for modern human origins* PhD thesis thesis, Arizona State University (2002).

66 Bailey, S. E. A morphometric analysis of maxillary molar crowns of Middle-Late Pleistocene hominins. *J Hum. Evol.* **47**, 183-198, doi:https://doi.org/10.1016/j.jhevol.2004.07.001 (2004).

67 Bailey, S. E. Beyond shovel-shaped incisors: Neandertal dental morphology in a comparative context. *Periodicum Biologorum* **108**, 253-267 (2006).

68 Villa, G. & Giacobini, G. Subvertical grooves of interproximal facets in neandertal posterior teeth. *American Journal of Physical Anthropology* **96**, 51-62, doi:10.1002/ajpa.1330960106 (1995).

69 Toussaint, M. in *The Scladina I-4A Juvenile Neandertal. Palaeoanthropology and Context* (eds M Toussaint & D. Bonjean) 233-306 (E.R.A.U.L, 2014).

70 Hammer, Ø., Harper, D. A. T. & Ryan, P. D. PAST: paleontological statistics software package for education and data analysis. *Palaeontologia Electronica* **4** (2001).

71 Turner, C. G. I., Nichol, C. R. & Scott, G. R. in *Advances in dental anthropology* (eds M.A. Kelley & C.S. Larsen) 13-31 (Wiley-Liss, 1991).

72 Martin, R. M. G., Hublin, J.-J., Gunz, P. & Skinner, M. M. The morphology of the enamel–dentine junction in Neanderthal molars: Gross morphology, non-metric traits, and temporal trends. *J Hum. Evol.* **103**, 20-44, doi:https://doi.org/10.1016/j.jhevol.2016.12.004 (2017).

73 Benazzi, S., Bailey, S. E. & Mallegni, F. Brief communication: A morphometric analysis of the neandertal upper second molar leuca I. *American Journal of Physical Anthropology* **152**, 300-305, doi:10.1002/ajpa.22355 (2013).

74 Gilbert, M. T. P., Bandelt, H.-J., Hofreiter, M. & Barnes, I. Assessing ancient DNA studies. *Trends in Ecology & Evolution* **20**, 541-544, doi:https://doi.org/10.1016/j.tree.2005.07.005 (2005).

75 Krause, J. *et al.* A Complete mtDNA Genome of an Early Modern Human from Kostenki, Russia. *Current Biology* **20**, 231-236, doi:https://doi.org/10.1016/j.cub.2009.11.068 (2010).

76 Pääbo, S. *et al.* Genetic Analyses from Ancient DNA. *Annual Review of Genetics* **38**, 645-679, doi:10.1146/annurev.genet.37.110801.143214 (2004).

77 Briggs, A. W. *et al.* Patterns of damage in genomic DNA sequences from a Neandertal. *Proceedings of the National Academy of Sciences* **104**, 14616-14621, doi:10.1073/pnas.0704665104 (2007).

78 Sawyer, S., Krause, J., Guschanski, K., Savolainen, V. & Pääbo, S. Temporal Patterns of Nucleotide Misincorporations and DNA Fragmentation in Ancient DNA. *PLOS ONE* **7**, e34131, doi:10.1371/journal.pone.0034131 (2012).

79 Meyer, M. *et al.* A mitochondrial genome sequence of a hominin from Sima de los Huesos. *Nature* **505**, 403-406, doi:10.1038/nature12788 (2014).

80 Green, R. E. *et al.* A Complete Neandertal Mitochondrial Genome Sequence Determined by High-Throughput Sequencing. *Cell* **134**, 416-426, doi:https://doi.org/10.1016/j.cell.2008.06.021 (2008).

81 Green, R. E. *et al.* A Draft Sequence of the Neandertal Genome. *Science* **328**, 710-722, doi:10.1126/science.1188021 (2010).

82 Fu, Q. *et al.* A Revised Timescale for Human Evolution Based on Ancient Mitochondrial Genomes. *Current Biology* **23**, 553-559, doi:10.1016/j.cub.2013.02.044 (2013).

83 Fu, Q. *et al.* Genome sequence of a 45,000-year-old modern human from western Siberia. *Nature* **514**, 445-449, doi:10.1038/nature13810 (2014).

84 Ermini, L. *et al.* Complete Mitochondrial Genome Sequence of the Tyrolean Iceman. *Current Biology* **18**, 1687-1693, doi:https://doi.org/10.1016/j.cub.2008.09.028 (2008).

85 Fu, Q. *et al.* DNA analysis of an early modern human from Tianyuan Cave, China. *Proceedings of the National Academy of Sciences* **110**, 2223-2227, doi:10.1073/pnas.1221359110 (2013).

86 Gilbert, M. T. P. *et al.* Paleo-Eskimo mtDNA Genome Reveals Matrilineal Discontinuity in Greenland. *Science* **320**, 1787-1789, doi:10.1126/science.1159750 (2008).

87 Hajdinjak, M. *et al.* Reconstructing the genetic history of late Neanderthals. *Nature* **555**, 652, doi:10.1038/nature26151 (2018).

88 Peyrégne, S. *et al.* Nuclear DNA from two early Neandertals reveals 80,000 years of genetic continuity in Europe. *Science Advances* **5**, eaaw5873, doi:10.1126/sciadv.aaw5873 (2019).

89 Posth, C. *et al.* Deeply divergent archaic mitochondrial genome provides lower time boundary for African gene flow into Neanderthals. *Nature Communications* **8**, 16046, doi:10.1038/ncomms16046 (2017).

90 Briggs, A. W. *et al.* Targeted Retrieval and Analysis of Five Neandertal mtDNA Genomes. *Science* **325**, 318-321, doi:10.1126/science.1174462 (2009).

91 Brown, S. *et al.* Identification of a new hominin bone from Denisova Cave, Siberia using collagen fingerprinting and mitochondrial DNA analysis. *Scientific Reports* **6**, 23559, doi:10.1038/srep23559 (2016).

92 Douka, K. *et al.* Age estimates for hominin fossils and the onset of the Upper Palaeolithic at Denisova Cave. *Nature* **565**, 640-644, doi:10.1038/s41586-018-0870-z (2019).

93 Gansauge, M.-T. & Meyer, M. Selective enrichment of damaged DNA molecules for ancient genome sequencing. *Genome Research* **24**, 1543-1549, doi:10.1101/gr.174201.114 (2014).

94 Rougier, H. *et al.* Neandertal cannibalism and Neandertal bones used as tools in Northern Europe. *Scientific Reports* **6**, 29005, doi:10.1038/srep29005 (2016).

95 Skoglund, P. *et al.* Separating endogenous ancient DNA from modern day contamination in a Siberian Neandertal. *Proceedings of the National Academy of Sciences* **111**, 2229-2234, doi:10.1073/pnas.1318934111 (2014).

96 Slon, V. *et al.* Neandertal and Denisovan DNA from Pleistocene sediments. *Science* **356**, 605-608, doi:10.1126/science.aam9695 (2017).

97 Krause, J. *et al.* The complete mitochondrial DNA genome of an unknown hominin from southern Siberia. *Nature* **464**, 894, doi:10.1038/nature08976 (2010).

98 Reich, D. *et al.* Genetic history of an archaic hominin group from Denisova Cave in Siberia. *Nature* **468**, 1053, doi:10.1038/nature09710 (2010).

99 Sawyer, S. *et al.* Nuclear and mitochondrial DNA sequences from two Denisovan individuals. *Proceedings of the National Academy of Sciences* **112**, 15696-15700, doi:10.1073/pnas.1519905112 (2015).

100 Horai, S. *et al.* Man's place in hominoidea revealed by mitochondrial DNA genealogy. *Journal of Molecular Evolution* **35**, 32-43, doi:10.1007/bf00160258 (1992).

101 Katoh, K. & Standley, D. M. MAFFT Multiple Sequence Alignment Software Version 7: Improvements in Performance and Usability. *Molecular Biology and Evolution* **30**, 772-780, doi:10.1093/molbev/mst010 (2013).

102 Darriba, D., Taboada, G. L., Doallo, R. & Posada, D. jModelTest 2: more models, new heuristics and parallel computing. *Nature Methods* **9**, 772, doi:10.1038/nmeth.2109 (2012).

103 Kumar, S., Stecher, G. & Tamura, K. MEGA7: Molecular Evolutionary Genetics Analysis Version 7.0 for Bigger Datasets. *Molecular Biology and Evolution* **33**, 1870-1874, doi:10.1093/molbev/msw054 (2016).

104 Schliep, K. P. phangorn: phylogenetic analysis in R. *Bioinformatics* **27**, 592-593, doi:10.1093/bioinformatics/btq706 (2010).

105 Bouckaert, R. *et al.* BEAST 2: A Software Platform for Bayesian Evolutionary Analysis. *PLOS Computational Biology* **10**, e1003537, doi:10.1371/journal.pcbi.1003537 (2014).

106 Shapiro, B. *et al.* A Bayesian Phylogenetic Method to Estimate Unknown Sequence Ages. *Molecular Biology and Evolution* **28**, 879-887, doi:10.1093/molbev/msq262 (2010).

107 Baele, G., Lemey, P. & Vansteelandt, S. Make the most of your samples: Bayes factor estimators for high-dimensional models of sequence evolution. *BMC Bioinformatics* **14**, 85, doi:10.1186/1471-2105-14-85 (2013).

108 Lyman, R. L. *Quantitative Paleozoology*. (Cambridge University Press, 2008).

109 Vereshchagin, N. & Baryshnikov, G. in *Paleoecology of Beringia* (eds D.M. Hopkins, Ch.E. Schweiger, J.V. Matthews, & S.B. Young) 267-279 (Academic Press, 1982).

110 Kahlke, R.-D. *The History of the Origin, Evolution and Dispersal of the Late Pleistocene Mammuthus–Coelodonta Faunal Complex in Eurasia (Large Mammals)*. 1-219 (Fenske Companies, 1999).

111 Stefaniak, K. *Neogene and Quaternary Cervidae from Poland*. 204 (Institute of Systematics and Evolution of Animals Polish Academy of Sciences, 2015).

112 Ratajczak, U. *et al.* Quaternary skulls of the saiga antelope from Eastern Europe and Siberia: Saiga borealis versus Saiga tatarica – One species or two? *Quatern. Int.* **420**, 329-347, doi:https://doi.org/10.1016/j.quaint.2015.09.040 (2016).

113 Baca, M. *et al.* Retreat and extinction of the Late Pleistocene cave bear (Ursus spelaeus sensu lato). *The Science of Nature* **103**, 92, doi:10.1007/s00114-016-1414-8 (2016).

114 Nadachowski, A. *et al.* *Late Pleistocene environment of the Częstochowa Upland (Poland) estimated from faunistic evidence of archaeological cave sites*. 112 (Institute of Systematics and Evolution of Animals, Polish Academy of Sciences, 2009).

115 Socha, P. Rodent palaeofaunas from Biśnik Cave (Kraków-Częstochowa Upland, Poland): Palaeoecological, palaeoclimatic and biostratigraphic reconstruction. *Quatern. Int.* **326-327**, 64-81, doi:https://doi.org/10.1016/j.quaint.2013.12.027 (2014).

116 Stefaniak, K., Socha, P., Nadachowski, A. & Tomek, T. in *Karst of the Częstochowa Upland and the Eastern Sudetes: palaeoenvironments and protection* Vol. 56 (eds K. Stefaniak, P. Socha, & A. Tyc) 85-144 (Studies of the Faculty of Earth Sciences, University of Silesia, 2009).

117 Baca, M. *et al.* Highly divergent lineage of narrow-headed vole from the Late Pleistocene Europe. *Scientific Reports* **9**, 17799, doi:10.1038/s41598-019-53937-1 (2019).

118 Picin, A. & Cascalheira, J. in *Short-Term Occupations in Paleolithic Archaeology: Definition and Interpretation* (eds J. Cascalheira & A. Picin) 1-15 (Springer International Publishing, 2020).

119 Picin, A. *et al.* Neanderthal mobile toolkit in short-term occupations at Teixoneres Cave (Moia, Spain). *Journal of Archaeological Science: Reports* **29**, 102165, doi:https://doi.org/10.1016/j.jasrep.2019.102165 (2020).

120 Picin, A. in *Short-Term Occupations in Paleolithic Archaeology: Definition and Interpretation* (eds J. Cascalheira & A. Picin) 73-103 (Springer International Publishing, 2020).

121 Kozłowski, S. K. E. *Wylotne and Zwierzyniec: Paleolithic sites in southern Poland*. (Polish Academy of Arts and Sciences, 2006).

122 Chmielewski, W. in *Paleolit i Mezolit 1975* 9-58 (1975).

123 Kozłowski, J. K. in *Studies on Raj cave near Kielce (Poland) and its deposits* 61-132 (Folia Quaternaria 41, 1972).

124 Sachse-Kozłowska, E. & Kozłowski, S. K. *Piekary près de Cracovie (Pologne) complexe de sites Paléolithiques*. (PAU, 2004).

125 Schild, R. *The killing fields of Zwolen. A Middle Paleolithic kill-butchery site in Central Europe*. (Institute of Archaeology and Ethnology Polish Academy of Sciences, 2005).

126 Valde-Nowak, P. *et al.* Late Middle Palaeolithic occupations in Ciemna Cave, southern Poland. *Journal of Field Archaeology* **41**, 193-210, doi:10.1080/00934690.2015.1101942 (2016).

127 Valde-Nowak, P. & Nadachowski, A. Micoquian assemblage and environmental conditions for the Neanderthals in Obłazowa Cave, Western Carpathians, Poland. *Quatern. Int.* **326–327**, 146-156, doi:http://dx.doi.org/10.1016/j.quaint.2013.08.057 (2014).

128 Golovanova, L. V. & Doronichev, V. B. The Middle Paleolithic of the Caucasus. *Journal of World Prehistory* **17**, 71-140, doi:10.1023/a:1023960217881 (2003).

129 Doronicheva, E. *et al.* Hadjoh-2: A Middle Paleolithic Workshop-Camp in Northwestern Caucasus. *Archaeology, Ethnology & Anthropology of Eurasia* **46**, 16-26, doi:https://doi.org/10.17746/1563-0110.2018.46.1.016-026 (2018).

130 Hoffecker, J. F., Baryshnikov, G. & Potapova, O. Vertebrate remains from the Mousterian site of Il'skaya I (northern Caucasus, U.S.S.R.): New analysis and interpretation. *J. Archaeol. Sci.* **18**, 113-147, doi:https://doi.org/10.1016/0305-4403(91)90043-O (1991).

131 Golovanova, L. V. & Doronichev, V. B. *Excavations of the Paleolithic site of Baranakha-4 in Northern Caucasus*. 201 (IA RAN, 1997).

132 Liubin, V. in *Neandertal’tsi Gubskogo ushelia na Severnom Kavkaze* 151-164 (Meoti, 1994).

doi:http://dx.doi.org/10.1016/j.quascirev.2013.12.012 (2014).

133 Helmens, K. F. The Last Interglacial–Glacial cycle (MIS 5–2) re-examined based on long proxy records from central and northern Europe. *Quaternary Sci. Rev.* **86**, 115-143,

134 Reimer, P. J. et al. The IntCal20 Northern Hemisphere radiocarbon age calibration curve (0–55 cal kBP). Radiocarbon, 1-33, doi:10.1017/RDC.2020.41 (2020).

135 Ramsey, C. B. Bayesian analysis of radiocarbon dates. *Radiocarbon* **51**, 337-360, doi:https://doi.org/10.1017/S0033822200033865 (2009).

136 Nowaczewska, W. *et al.* The tooth of a Neanderthal child from Stajnia Cave, Poland. *J Hum. Evol.* **64**, 225-231, doi:http://dx.doi.org/10.1016/j.jhevol.2012.12.001 (2013).

137 Martinón-Torres, M., Bermúdez de Castro, J. M., Gómez-Robles, A., Prado-Simín, L. & Arsuaga, J. L. Morphological description and comparison of the dental remains from Atapuerca-Sima de los Huesos site (Spain). *J Hum. Evol.* **62**, 7-58 (2012).

138 Hershkovitz, I. *et al.* The earliest modern humans outside Africa. *Science* **359**, 456-459, doi:10.1126/science.aap8369 (2018).

139 Bermúdez de Castro, J. M. Dental remains from Atapuerca (Spain) I. Metrics. *J Hum. Evol.* **15**, 265-287, doi:https://doi.org/10.1016/S0047-2484(86)80054-9 (1986).

140 Bermúdez de Castro, J. M. The Atapuerca dental remains. New evidence (1987-1991 excavations) and interpretations. *J Hum. Evol.* **24**, 339-371, doi:https://doi.org/10.1006/jhev.1993.1027 (1993).

141 Murrill, R. I. A comparison of the Rhodesian and Petralona upper jaws in relation to other Pleistocene hominids. *Zeitschrift für Morphologie und Anthropologie* **66**, 176-187 (1975).

142 Pinilla, B. & Trinkaus, E. in *The People of Palomas. Neandertals from the Sima de las Palomas del Cabezo Gordo, Southeastern Spain* (eds Trinkaus E. & Walker MJ.) 89-104 (Texas A & M University Press, 2017).

143 Trinkaus, E. Dental remains from the Shanidar adult Neanderthals. *J Hum. Evol.* **7**, 369-382, doi:https://doi.org/10.1016/S0047-2484(78)80087-6 (1978).

144 McCown, T. D. & Keith, A. *The Stone Age of Mount Carmel. Vol. 2, The fossil human remains from the Levalloiso-Mousterian*. (The Clarendon Press, 1939).

145 Wolpoff, M. H. The Krapina dental remains. *American Journal of Physical Anthropology* **50**, 67-113, doi:10.1002/ajpa.1330500110 (1979).

146 White, T. D. *et al.* Pleistocene Homo sapiens from Middle Awash, Ethiopia. *Nature* **423**, 742-747 (2003).

147 Day, M. H. *Guide to Fossil Man*. (Cassell, 1986).

148 Matiegka, D. J. *L'Homme fossile de Předmostí en Moravie (Tchécoslovaquie). I. Les crânes*. (Nákl. České Akademie věd a umění, 1934).

149 Matiegka, D. J. The skull of the fossil man Brno III, and the cast of its interior. *Anthropologie (1923-1941)*, 90-107 (1929).

150 Sládek, V., Trinkaus, E., Hillson, S. W. & Holliday, T. W. *The people of the Pavlovian. Skeletal catalogue and osteometrics of the Gravettian fossil hominids from Dolni Vestonice and Pavlov. Dolni Vestonice Studie, Svazek 5.*, (Archeologický ústav AV ČR, 2000).

151 Frayer, D. W., Jelínek, J., Oliva, M. & Wolpoff, M. H. in *Early Modern Humans at the Moravian Gate: The Mladeč Caves and their Remains* (ed M. Teschler-Nicola) 185-272 (Springer Vienna, 2006).

152 Wolpoff, M. H., Frayer, D. W. & Jelínek, J. in *Early Modern Humans at the Moravian Gate: The Mladeč Caves and their Remains* (ed M. Teschler-Nicola) 273-340 (Springer Vienna, 2006).

153 Trinkaus, E., Buzhilova, A. P., Mednikova, M. a. B. & Dobrovolʹskai︠a︡, M. a. V. *The people of Sunghir: burials, bodies, and behavior in the earlier Upper Paleolithic*. (Oxford University Press, 2014).

154 Martinón-Torres, M. *et al.* Dental remains from Dmanisi (Republic of Georgia): Morphological analysis and comparative study. *J Hum. Evol.* **55**, 249-273, doi:https://doi.org/10.1016/j.jhevol.2007.12.008 (2008).

155 Reimer, P. J. *et al.* IntCal13 and Marine13 radiocarbon age calibration curves 0–50,000 years cal BP. *Radiocarbon* **55**, 1869-1887, doi:https://doi.org/10.2458/azu_js_rc.55.16947 (2013).

156 Bronk Ramsey, C. & Lee, S. Recent and planned developments of the program OxCal. *Radiocarbon* **55**, 720-730, doi:https://doi.org/10.1017/S0033822200057878 (2013).

157 Fewlass, H. *et al.* Direct radiocarbon dates of mid Upper Palaeolithic human remains from Dolní Věstonice II and Pavlov I, Czech Republic. *Journal of Archaeological Science: Reports* **27**, 102000, doi:https://doi.org/10.1016/j.jasrep.2019.102000 (2019).

158 Pinhasi, R., Higham, T. F. G., Golovanova, L. V. & Doronichev, V. B. Revised age of late Neanderthal occupation and the end of the Middle Paleolithic in the northern Caucasus. *Proceedings of the National Academy of Sciences* **108**, 8611-8616, doi:10.1073/pnas.1018938108 (2011).

159 Schmitz, R. W. *et al.* The Neandertal type site revisited: Interdisciplinary investigations of skeletal remains from the Neander Valley, Germany. *Proceedings of the National Academy of Sciences* **99**, 13342-13347, doi:10.1073/pnas.192464099 (2002).

160 Serre, D. *et al.* No Evidence of Neandertal mtDNA Contribution to Early Modern Humans. *PLOS Biology* **2**, e57, doi:10.1371/journal.pbio.0020057 (2004).

161 Semal, P. *et al.* New data on the late Neandertals: Direct dating of the Belgian Spy fossils. *American Journal of Physical Anthropology* **138**, 421-428, doi:10.1002/ajpa.20954 (2009).

162 Linnaeus, C. *Systema naturae per regna tria naturae, secundum classes, ordines, genera, species, cum characteribus, differentiis, synonymis, locis*. (Laurentii Salvii, 1758).

163 Rabeder, G., Hofreiter, M. & Withalm, G. The systematic position of the cave bear from Potočka zijalka (Slovenia). *Mitt. Komm. Quartärforsch. Österr. Akad. Wiss.*, 197-200 (2004).

164 Lesson, R. P. *Manuel de mammalogie, ou Historie naturelle des mammifères*. Vol. 1 (Roret libraire, 1827).

165 Linnaeus, C. *Systema naturae sive regna tria naturae, secundum classes, ordines, genera, species, cum characteribus, differentiis, synonymis, locis. Vol. I, No. 1, Edito duodecima, reformata*. (Laurentii Salvii, 1766).

166 Goldfuss, A. *Mikroskopische Beobachtungen über die Metamorphose des vegetabilischen und animalischen Lebens*. (Abhandlungen der Erlanger Societät. Band 1, 1810).

167 Schreber, J. C. *Die Säugetiere in Abbildungen nach der Natur mit Beschreibungen*. (Walther, 1977).

168 Goldfuss, A. *Ein Wort über die Bedeutung naturwissenschaftlicher Institute und über ihren Einfluss auf humane Bildung*. (1823).

169 Blumenbach, J. *Handbuch der naturgeschichte16*. 697 (1799).

170 Blumenbach, J. F. *Geschichte und Beschreibung der Knochen des menschlichen Körpers*. (Dieterich, 1807).

171 Boddaert, P. *Elenchus Animalium vol. 1*. 174 (1785).

172 Bojanus, H. *De uro nostrato ejusque sceleto commentatio, Bovis primigenii sceleto aucta*. (Nov. Act. Ac. Leopold., 1827).
